# Distinctive reproductive tract microbial characteristics associated with infertility in women: a systematic review and meta-analysis

**DOI:** 10.3389/fcimb.2026.1836100

**Published:** 2026-06-18

**Authors:** Zhi-Yong Xiao, Yu-Xin Zhang, Jia-Jia Liu, Xiao-Yan Zheng, Han Yang, Jie Wu, Jie Yang

**Affiliations:** 1 Acupuncture and Tuina School, Chengdu University of Traditional Chinese Medicine, Chengdu, Sichuan, China; 2 Hospital of Chengdu University of Traditional Chinese Medicine, Chengdu, Sichuan, China; 3 Division of Internal Medicine, Institute of Integrated Traditional Chinese and Western Medicine, West China Hospital, Sichuan University, Chengdu, Sichuan, China

**Keywords:** female infertility, meta-analysis, microbial dysbiosis, reproductive tract microbiota, α-diversity

## Abstract

**Objective:**

To evaluate α-diversity alterations, summarize microbial taxa reported to be associated with infertility, and examine site-specific differences in reproductive tract microbiota between infertile and fertile women via systematic review and meta-analysis.

**Data sources:**

Medline, Embase, the Cochrane Central Register of Controlled Trials (CENTRAL), and Web of Science were searched from inception to December 30, 2025, supplemented by clinical trial registries, conference proceedings, and manual reference screening.

**Study eligibility criteria:**

Observational studies (cross-sectional, case-control, cohort) including women 18–45 years with infertility versus healthy fertile controls, using high-throughput sequencing of reproductive tract microbiota, reporting α-diversity or taxa abundance. Excluded randomized controlled trials (RCTs), animals, reviews.

**Study appraisal and synthesis methods:**

PRISMA-compliant review. The Newcastle-Ottawa Scale (NOS) for quality, the Grading of Recommendations Assessment, Development, and Evaluation (GRADE) for evidence. Random-effects meta-analysis with standardized mean differences (SMD) and 95% credible interval (CrI); I² for heterogeneity; subgroup and sensitivity analyses by site.

**Results:**

Twenty-seven studies with 2174 participants were included. Infertile women had higher α-diversity (Shannon SMD 0.27 [0.10 to 0.44; I² = 40%], Simpson SMD 0.24 [0.01 to 0.47; I² = 52%], ACE SMD 0.43 [0.15 to 0.71; I² = 33%]). *Lactobacillus* abundance was lower (SMD -0.40 [-0.55 to -0.25; I² = 0%]), while *Prevotella* (SMD 0.48 [0.27 to 0.70; I² = 12%]) and *Burkholderia* (SMD 0.55 [0.33 to 0.77; I² = 0%]) were higher. Changes were prominent in vaginal samples, and β-diversity differed in 81% of studies.

**Conclusions:**

Infertile women show site-specific microbiota patterns, including reduced *Lactobacillus* and elevated opportunistic pathogens in the reproductive tract, especially vaginally. Given the low to very low certainty of largely observational evidence, high-quality prospective studies are needed to validate these associations and clarify causality.

**Systematic Review Registration:**

https://www.crd.york.ac.uk/prospero/, identifier CRD420251275219.

## 1 Introduction

The female reproductive tract primarily comprises the external vulva and internal structures, including the vagina, cervix, uterus, and fallopian tubes. These elements are anatomically interconnected, establishing a contiguous pathway that supports gamete transport, fertilization, and embryo implantation. Pathological changes within the reproductive system elevate the risk of female infertility, encompassing conditions such as bacterial vaginosis, fallopian tube anomalies, ovarian disorders, and uterine malformations (Penzias et al., 2021; Ravel et al., 2021; Bonavina and Taylor, 2022). This emphasizes the critical role of the reproductive tract in achieving successful reproduction. It is estimated that in 2021, the global prevalence of female infertility affected 110 million women, with projections suggesting a sustained increase in incidence over the next three decades (Liu et al., 2025). The societal stigma associated with infertility not only imposes psychological burdens on women but also leads to declining birth rates, which can profoundly impact national population structures, economies, and environments (Liu et al., 2025).

Emerging evidence substantiates a strong association between the reproductive tract microbiota and female reproductive health. In the majority of healthy women, the microbial composition of the reproductive tract is predominantly characterized by *Lactobacillus* species (Ma et al., 2012), comprising over 90% of the community in certain instances (Kyono et al., 2018; Ichiyama et al., 2021). This dominance confers protection potentially through the synthesis of lactic acid, which maintains an acidic pH environment to suppress the proliferation of opportunistic pathogens (Chen et al., 2017). In contrast, microbial dysbiosis has been implicated in adverse reproductive outcomes, including infertility. As the most accessible site for sampling within the internal reproductive tract, the vagina has yielded substantial insights into microbial imbalances among infertile women. Relative to fertile women of reproductive age, the vaginal microbiota in infertile individuals often demonstrates elevated α-diversity, marked by a diminished relative abundance of *Lactobacillus* and increased prevalence of genera such as *Gardnerella*, *Prevotella*, and *Atopobium* (Huang et al., 2019; Mohammadi et al., 2025). However, several studies have reported inconsistent findings, including reduced α-diversity in the vaginal microbiota of infertile women (Ichiyama et al., 2021), along with greater enrichment of *Lactobacillus* (Barinova et al., 2021; Chen et al., 2021; Hasan et al., 2024). These conflicting results highlight the need for a systematic synthesis of existing evidence. Research on the cervical and endometrial microbiomes is comparatively limited. Similar to vaginal microbiome studies, alterations in multiple microorganisms associated with bacterial vaginosis (BV), such as *Gardnerella vaginalis*, *Atopobium vaginae*, and *Prevotella bivia*, may be related to uterine cavity and pelvic inflammatory diseases (Fernández et al., 2021; Ravel et al., 2021). Nonetheless, the linkage to reproductive outcomes remains inconclusive (Toson et al., 2022).

To address these inconsistencies, we will conduct a meta-analysis of available evidence with three primary objectives: (1) to determine how α-diversity of the reproductive tract microbiota is altered in infertile women; (2) to identify microbial taxa associated with infertility in observational studies; and (3) to examine whether these microbial characteristics differ by anatomical site. This study aims to provide robust, consolidated evidence characterizing the reproductive tract microbiota in female infertility.

## 2 Methods

This systematic review and meta-analysis were conducted in accordance with the Preferred Reporting Items for Systematic Reviews and Meta-Analyses (PRISMA) guidelines(Cochrane Handbook for Systematic Reviews of Interventions version 6.5 (updated August 2024), (2024) (see **Supplementary Table 8**) and the Meta-analysis of Observational Studies in Epidemiology (MOOSE) reporting guidelines (Stroup et al., 2000). The protocol was prospectively registered on PROSPERO with the number (CRD420251275219).

### 2.1 Search strategy and study sources

We systematically searched Medline, Embase, the Cochrane Central Register of Controlled Trials (CENTRAL), and Web of Science from their inception up to 30 December 2025. The search strategy combined Medical Subject Headings (MeSH) terms and keywords related to female infertility and reproductive tract microbiome. Detailed search strategies are provided in **Supplementary Table 1**. To minimize publication bias, we also searched for unpublished or ongoing studies through clinical trial registries (e.g., ClinicalTrials.gov) and conference proceedings. Manual screening of reference lists supplemented the electronic search.

### 2.2 Eligibility criteria and study selection

This study adhered to the PICOS (Participants, Interventions, Comparators, Outcomes, Study design) framework, including studies that met the following criteria. (1) Study design: Observational studies, including cross-sectional, case-control, and cohort designs; randomized controlled trials, animal studies, case reports, reviews, editorials, and conference abstracts without full text were excluded. (2) Participants: Women aged 18–45 years diagnosed with infertility, defined as the inability to achieve pregnancy after 12 months or more of regular unprotected sexual intercourse (Penzias et al., 2021). If the study did not explicitly state this definition, women who underwent assisted reproductive technologies (ART) due to female factors were also considered infertile. Women seeking treatment for male infertility were excluded. (3) Interventions: Studies utilizing high-throughput sequencing for microbial profiling of the reproductive tract, such as 16S rRNA sequencing or metagenomics. Studies limited to cell culture were excluded. (4) Comparators: Healthy women of reproductive age, matched where possible for age, ethnicity, and other confounders (e.g., body mass index, hormonal status, sexual activity). Health was defined as having a history of natural conception and delivery or no identified factors impeding pregnancy upon examination. Women who had conceived via ART and those who were currently pregnant were excluded. (5) Outcomes: reports of microbiota diversity and relative abundance. Specifically, primary indicators included α-diversity (e.g., Shannon index, Simpson index, and Chao1 index) and relative abundance of taxa, while secondary indicators comprised β-diversity, with studies eligible for inclusion if they reported at least one of these measures.

After removing duplicates, titles and abstracts were reviewed to identify studies meeting the inclusion criteria. Full texts of selected studies were retrieved and screened against predefined criteria. Exclusion decisions were documented for transparency and reproducibility. Screening was independently conducted by two authors, with disagreements resolved through consultation with a third author.

### 2.3 Data extraction

Two authors independently extracted data from the included studies, including study ID (first author’s surname and publication year), country, inclusion and exclusion criteria, sample size, sample source, sequencing technology, age, body mass index (BMI), and reported outcomes required for this study. Results for α-diversity and microbiota relative abundance were recorded as mean and standard deviation (SD). If data were presented only in graphical formats like box plots and attempts to obtain raw data from authors failed, values for variables of interest were extracted using WebPlotDigitizer (see **Supplementary Table 9**), and estimated using standardized methods(Cochrane Handbook for Systematic Reviews of Interventions version 6.5 (updated August 2024), (2024). Disagreements in data extraction were resolved by consensus or by consulting a third author.

### 2.4 Risk of bias assessment

The methodological quality of the included studies was independently assessed by two authors using the Newcastle-Ottawa Scale (NOS). The NOS evaluates study quality based on three domains: selection of study groups, comparability of groups, and ascertainment of the outcome of interest. Studies were categorized as high quality, moderate quality, or low quality. Furthermore, the Grading of Recommendations Assessment, Development, and Evaluation (GRADE) instrument was applied to rate the quality of evidence.

### 2.5 Statistical analysis

The meta-analysis was conducted using Review Manager 5.4.1 software. Data were pooled using a random-effects model, with results expressed as standardized mean differences (SMD) and 95% credible interval (CrI). Forest plots were generated to display individual study results alongside the overall pooled estimate. Statistical heterogeneity was evaluated with Cochran’s Q test and quantified using the I^2^ statistic. If I^2^ equals 40% or greater, it is considered to indicate moderate heterogeneity or high heterogeneity.(Cochrane Handbook for Systematic Reviews of Interventions version 6.5 (updated August 2024), (2024) Sensitivity analyses were performed to assess the effect of each study on pooled outcomes by sequentially excluding individual studies. For indicators with 10 or more studies, funnel plots were used to explore publication bias. Meta-regression analyses were performed for key variables, including age, geographic region, baseline BMI, ART history, and sample site. We conducted subgroup analyses based on whether the samples were derived from the vagina, cervix, or endometrium.

## 3 Results

### 3.1 Search results

A total of 5129 articles were identified through a comprehensive database search, supplemented by manual screening. After a rigorous screening process, twenty-seven studies involving 2174 participants met the inclusion criteria (Dai et al., 2016; Moreno et al., 2016; Campisciano et al., 2017; Graspeuntner et al., 2018; Kyono et al., 2018; Wee et al., 2018; Huang et al., 2019; Vladislavovna et al., 2020; Zhao et al., 2020, 2025; Barinova et al., 2021; Chen et al., 2021, 2025a, 2025b; Fernández et al., 2021; Ichiyama et al., 2021; Manzoor et al., 2021; Sun et al., 2021; Patel et al., 2022; Dong et al., 2023; Chopra et al., 2024; Haahr et al., 2024; Hasan et al., 2024; Su et al., 2024; Aldhalimi et al., 2025; Gao et al., 2025; Mohammadi et al., 2025) with twenty-one studies incorporated into this meta-analysis. **Supplementary Figure 1** shows the selection process, and **Supplementary Table 2** is the reasons for the final exclusion of the probable literature.

These studies were conducted across 13 countries and published between 2016 and 2025. In eighteen studies, the healthy control groups had a clear fertility history. Four studies simultaneously collected samples from the vagina, cervix, and endometrium. Six studies and seventeen studies collected samples from two and one site, respectively. **Table 1** presents the main characteristics of the included studies, with additional characteristics available in **Supplementary Table 3**.

**Table 1:** Main characteristics of studies included.

ID	Disease	Control	Samplesize (Infertility/Control)	Age(mean ± sd)	Indicators
Wee et al., 2018 ^abc^	Infertility or IVF	Parous women	31(15/16)	40.26 (mean)	Relative abundance, α-diversity
Chen et al., 2021 ^a^	Infertility	Healthy women	21(14/7)	33.86 ± 4.01	Relative abundance, Observed species, Shannon, Simpson, Chao1, ACE, Good’s coverage, β-diversity
Hasan et al., 2024 ^a^	Infertility	Parous women	10(5/5)	NA	Observed species, Shannon, Simpson, Chao1, ACE, Jackknife, Phylogenetic diversity, β-diversity
Kyono et al., 2018 ^ac^	Infertility or IVF	Parous women	109(102/7)	36.17 ± 4.51	Relative abundance
Ichiyama et al., 2021 ^ac^	IVF	Healthy women	166(145/21)	37.5 ± 4.66	Relative abundance, Shannon, Chao1, β-diversity
Fernández et al., 2021 ^ab^	IVF	Parous women	37(23/14)	38.67 ± 1.53	Relative abundance, Shannon, Simpson, β-diversity
Manzoor et al., 2021 ^#^	Infertility	Parous women	30(16/14)	28.25 ± 5.47	Relative abundance, Observed species, Shannon, Faith PD, Evennnes, β-diversity
Zhao et al., 2020 ^a^	IVF	Healthy women	80(30/50)	30.72 ± 7.27	Relative abundance, Chao1, β-diversity
Sun et al., 2021 ^ac^	Infertility	Healthy women	30(15/15)	NA	Relative abundance
Huang et al., 2019 ^a^	Infertility	Parous women	30(15/15)	30.93 ± 5.27	Relative abundance, Shannon, Simpson, Chao1, ACE, β-diversity
Chen et al., 2025a_1^a^	Infertility	Parous women	22(15/7)	NA	Relative abundance
Patel et al., 2022 ^a^	IVF	Healthy women	24(16/8)	30.96 ± 4.78	Relative abundance
Zhao et al., 2025 ^a^	IVF	Parous women	114(77/37)	31.41 ± 4.95	Relative abundance, Shannon, Simpson, β-diversity
Moreno et al., 2016 ^ac^	IVF	Parous women	55(35/22)	NA	Relative abundance, Shannon, β-diversity
Mohammadi et al., 2025 ^abc^	IVF	Healthy women	50(40/10)	NA	Relative abundance
Gao et al., 2025 ^c^	IVF	Healthy women	36(17/19)	32.66 ± 5.17	Relative abundance, Shannon, Chao1, β-diversity
Vladislavovna et al., 2020 ^c^	IVF	Healthy women	42(22/20)	31.61 ± 4.59	Relative abundance
Graspeuntner et al., 2018 ^b^	Infertility	Parous women	136(47/89)	NA	Relative abundance, Simpson
Aldhalimi et al., 2025 ^a^	Infertility	Parous women	310(210/100)	30.51 ± 5.08	Shannon, Simpson
Chopra et al., 2024 ^a^	Infertility	Parous women	80(40/40)	25.72 ± 5.39	Relative abundance, Observed species, Shannon, Simpson, Chao1, ACE, Fisher, β-diversity
Dai et al., 2016 ^a^	Infertility	Parous women	80(40/40)	30.73 ± 5.64	Relative abundance, Shannon, β-diversity
Campisciano et al., 2017 ^a^	Infertility	Parous women	96(27/69)	35.53	Relative abundance, Chao1, Simpson, β-diversity
Haahr et al., 2024 ^abc^	IVF	Healthy women	53(27/26)	30.08 ± 4	Relative abundance, β-diversity
Chen et al., 2025b_2^a^	IVF	Parous women	294(194/100)	31.9 ± 4.71	Relative abundance, Observed species, Shannon, Chao1, ACE, β-diversity
Dong et al., 2023 ^ab^	IVF	Parous women	32(22/10)	32.15 ± 4.7	Relative abundance, Shannon, Chao1, ACE, Simpson, Observed species, β-diversity
Barinova et al., 2021 ^c^	IVF	Parous women	35(20/15)	32.74 ± 4.96	Relative abundance
Su et al., 2024 ^abc^	IVF	Parous women	171(154/17)	NA	Relative abundance, Faith, β-diversity

**Table 1** presents the primary characteristics of the included studies, including ID (composed of the author and year of publication), sample source (a, b, c respectively denoting samples from the vagina, cervix, and endometrium), sample size, age, and reported indicators. In the Disease column, IVF indicates that the infertile female patients in the corresponding study are undergoing or will undergo assisted reproductive technology IVF, otherwise marked as Infertility; in the control column, Parous women indicates that the inclusion criteria for the control group in the corresponding study encompass a history of parity, otherwise marked as healthy women, denoting no history of parity but no evident factors detrimental to fertility upon examination. BMI, Body mass index; IVF, *In vitro* fertilization; NA indicates not mentioned.

### 3.2 α-diversity

**Figure 1:**
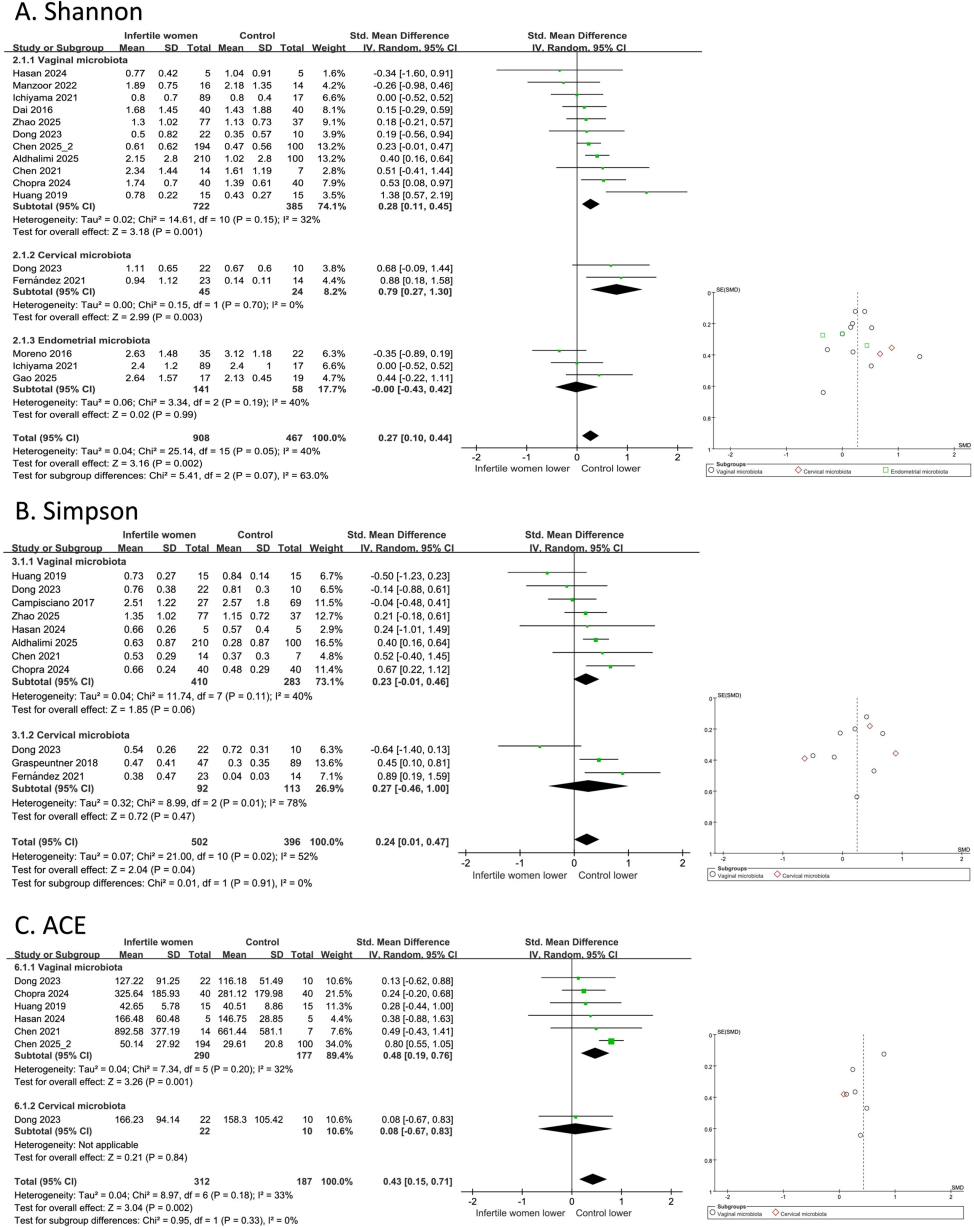
Results of α-diversity analysis. Displays the results for three α-diversity metrics of reproductive tract microbiota and the corresponding funnel chart, with panels **(A–C)** representing the Shannon index, Simpson index, and ACE index, respectively. The figure highlights that α-diversity differences varied across indices and anatomical sites, supporting a site-specific interpretation of microbial diversity patterns.

Nineteen studies (Dai et al., 2016; Moreno et al., 2016; Campisciano et al., 2017; Graspeuntner et al., 2018; Wee et al., 2018; Huang et al., 2019; Zhao et al., 2020, 2025; Chen et al., 2021, 2025b; Fernández et al., 2021; Ichiyama et al., 2021; Manzoor et al., 2021; Dong et al., 2023; Chopra et al., 2024; Hasan et al., 2024; Su et al., 2024; Aldhalimi et al., 2025; Gao et al., 2025) (19/27, 70.37%) provided results for at least one α-diversity metric. Our analysis revealed that, compared to healthy controls, the reproductive tract microbiota in infertile women exhibited significant elevations in multiple α-diversity indices. The Shannon index, analyzed in fourteen studies (involving 908 reproductive tract samples from infertile women and 467 from control women), was significantly higher in infertile women (SMD 0.27; 95% CrI 0.10 to 0.44; I² = 40%; **Figure 1A**). Subgroup analysis indicated significant elevations in the Shannon index for vaginal microbiota (SMD 0.28; 95% CrI 0.11 to 0.45; I² = 32%; **Figure 1A**) and cervical microbiota (SMD 0.79; 95% CrI 0.27 to 1.30; I² = 0%; **Figure 1A**) in infertile women, but no significant differences were observed in endometrial samples. The Simpson index, from ten studies (involving 502 reproductive tract samples from infertile women and 396 from control women), also showed significant elevation in infertile women (SMD 0.24; 95% CrI 0.01 to 0.47; I² = 52%; **Figure 1B**). Subgroup analysis revealed no significant differences in vaginal or cervical samples, with a lack of studies on endometrial samples. Six studies analyzed the ACE index (involving 312 reproductive tract samples from infertile women and 187 from control women), demonstrating a significant elevation in infertile women compared to controls (SMD 0.43; 95% CrI 0.15 to 0.71; I² = 33%; **Figure 1C**). Subgroup analysis showed a significant elevation in the ACE index for vaginal microbiota in infertile women (SMD 0.48; 95% CrI 0.19 to 0.76; I² = 32%; **Figure 1B**), but no significant differences in cervical samples, with a lack of studies on endometrial samples.

**Figure 2:**
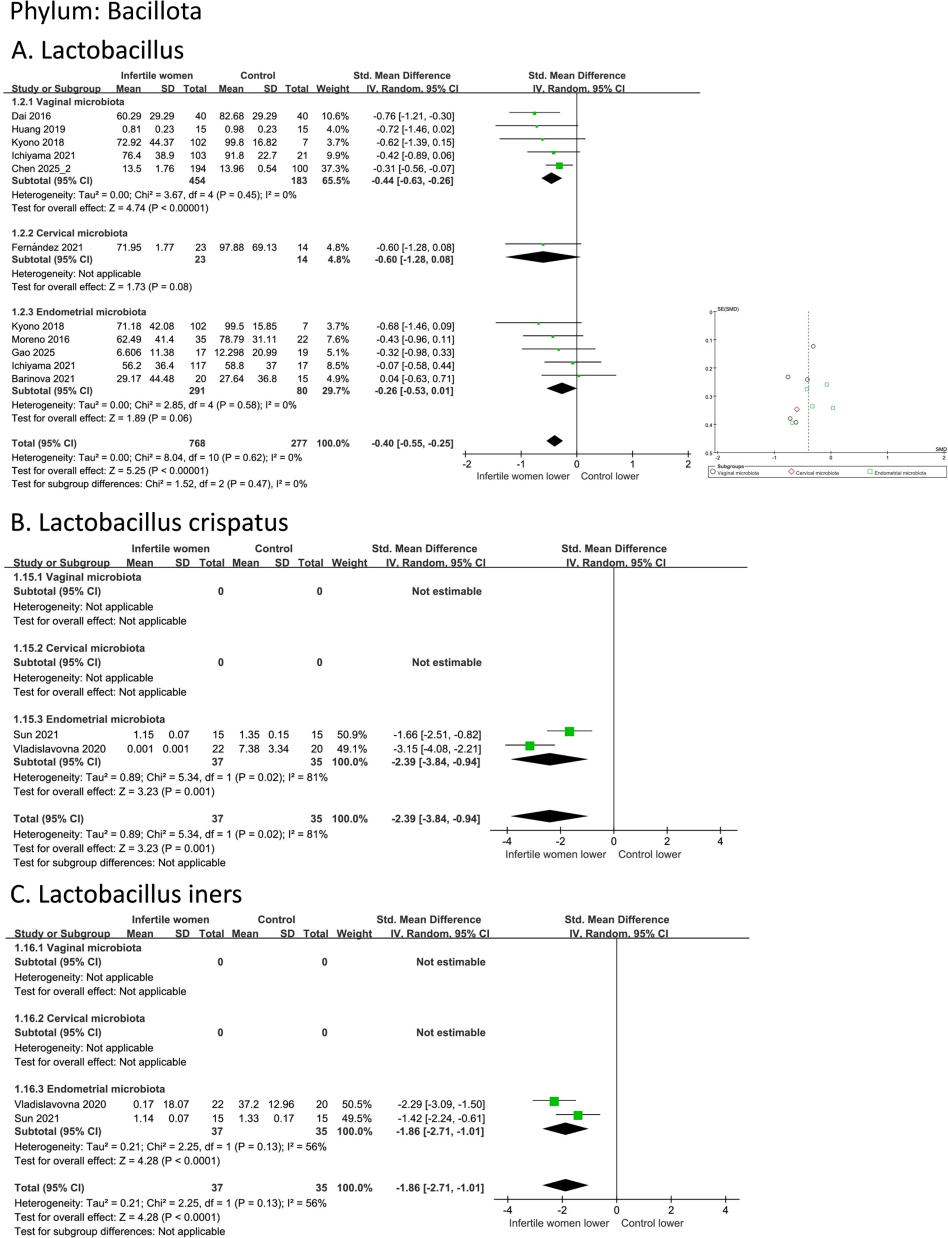
The difference in the relative abundance of microbiota. Displays the forest plot results and corresponding funnel plots for *Lactobacillus*, *Lactobacillus crispatus*, and *Lactobacillus iners* in panels **(A–C)**, respectively. The figure illustrates taxa-level abundance patterns, particularly reduced *Lactobacillus*-related taxa, while showing that these differences should be interpreted in relation to anatomical site and study-level variability.

No significant differences were observed in the Observed index (from 6 studies; 313 infertile women samples and 186 control samples) or Chao1 index (from 10 studies; 564 infertile women samples and 359 control samples) between infertile women and healthy controls (see **Supplementary Figure 2**). Funnel plots indicated no potential publication bias for the Shannon and Simpson indices, but possible publication bias for the ACE and Chao1 indices (see **Figure 2** and **Supplementary Figure 2**).

Overall, the forest plots suggest that α-diversity tended to be higher in infertile women, but this pattern was not uniform across indices or anatomical sites. The Shannon and ACE indices showed more consistent increases in vaginal samples, whereas evidence from cervical and endometrial samples was limited or inconsistent.

### 3.3 Relative abundance of microbes

We performed a meta-analysis on taxa reported in at least two studies to identify microbial taxa associated with infertility, incorporating eleven studies (11/27, 40.74%) (Dai et al., 2016; Moreno et al., 2016; Kyono et al., 2018; Huang et al., 2019; Vladislavovna et al., 2020; Barinova et al., 2021; Fernández et al., 2021; Ichiyama et al., 2021; Sun et al., 2021; Chen et al., 2025b; Gao et al., 2025). This analysis included 16 taxa at various taxonomic levels: phylum-level Bacillota; genus-level *Lactobacillus*, *Atopobium*, *Gardnerella*, *Prevotella*, *Delftia*, *Enterococcus*, *Megasphaera*, *Schlegelella*, *Ralstonia*, *Burkholderia*, *Pseudomonas*, *Bifidobacterium*, and *Escherichia-Shigella*; and species-level *Lactobacillus crispatus* and *Lactobacillus iners*.

**Figure 3:**
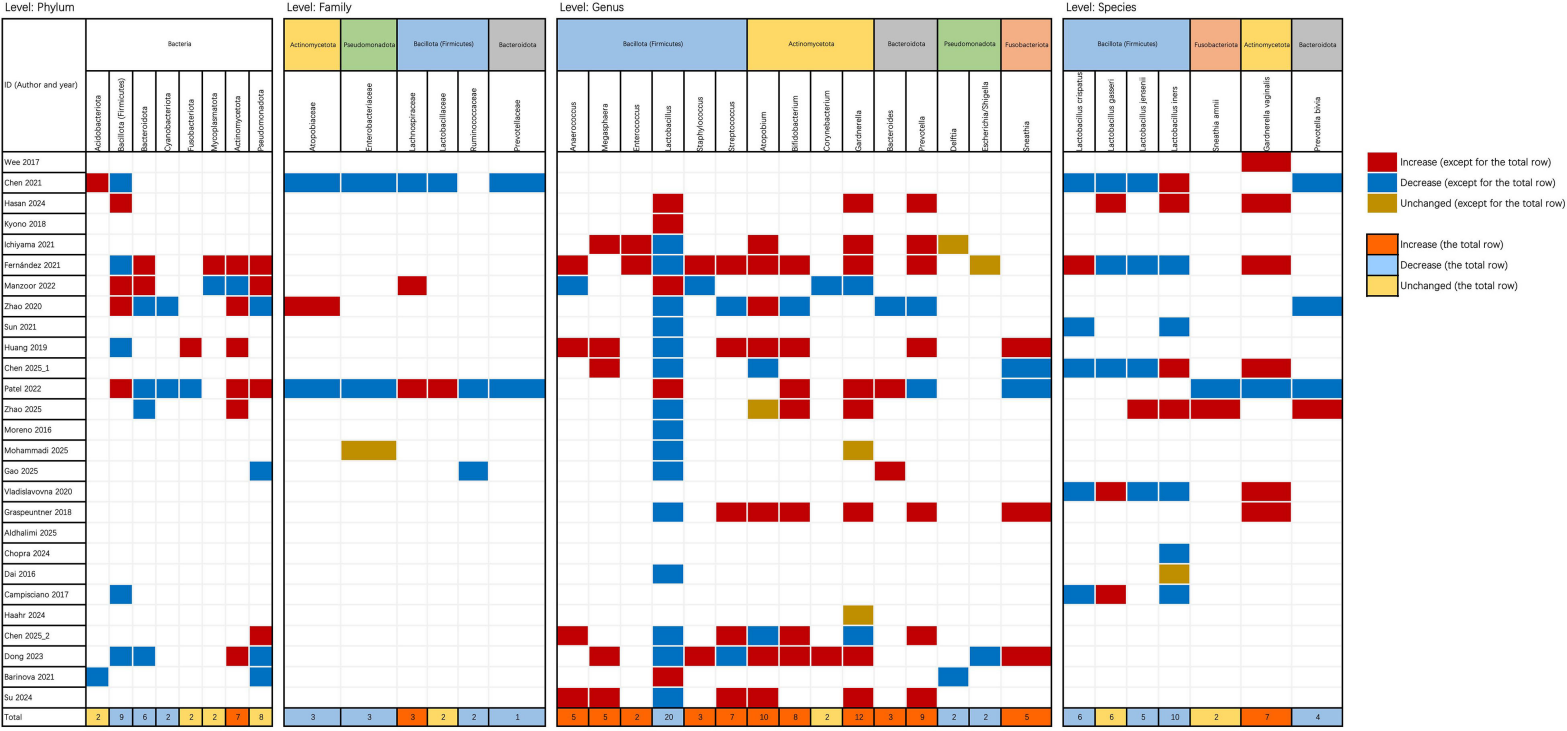
Changes in microbial communities at various taxonomic levels in the reproductive tract of infertile women. Summarizes the change of microbial taxa reported in 2 or more studies. Columns represent study IDs, rows represent taxa at different levels, with color blocks: red for increase, blue for decrease, brown for no change. The Total row shows majority study results, with orange for increase, blue for decrease, yellow for no change; numbers in blocks indicate the count of corresponding references. This figure provides an overview of recurring taxa-level patterns and helps distinguish consistent findings from study-specific signals.

Our analysis found six microbial taxa showing statistically significant observational associations with infertility. Specifically, the relative abundance of genus-level *Lactobacillus* was significantly lower in infertile women compared to controls (SMD -0.40; 95% CrI -0.55 to -0.25; I² = 0%; **Figure 2A**), based on nine studies involving 768 samples from infertile women and 277 from controls. Subgroup analysis showed a significant decrease in vaginal *Lactobacillus* relative abundance in infertile women (SMD -0.44; 95% CrI -0.63 to -0.26; I² = 0%; **Figure 2A**), with no notable differences in cervical or endometrial samples. The relative abundances of *Lactobacillus crispatus* (SMD -2.39; 95% CrI -3.84 to -0.94; I² = 81%; **Figure 2B**) and *Lactobacillus iners* (SMD -1.86; 95% CrI -2.71 to -1.01; I² = 56%; **Figure 2C**) were also significantly decreased, based on 37 endometrial samples from infertile women and 35 from controls. Additionally, genus-level *Enterococcus* was significantly decreased in the reproductive tract of infertile women (SMD -0.53; 95% CrI -1.03 to -0.04; I² = 0%; see **Supplementary Figure 3**; based on 38 samples from infertile women and 29 from controls). Conversely, genus-level *Prevotella* (SMD 0.48; 95% CrI 0.27 to 0.70; I² = 12%; see **Supplementary Figure 4**; based on 437 samples from infertile women and 152 from controls) and *Burkholderia* (SMD 0.55; 95% CrI 0.33 to 0.77; I² = 0%; see **Supplementary Figure 5**; based on 311 samples from infertile women and 117 from controls) were significantly increased. **Figure 3** summarizes the microbial community change results that were reported in 2 or more original studies but could not be subjected to meta-analysis. Due to data limitations, a funnel plot was generated only for genus-level *Lactobacillus*, indicating potential publication bias.

The taxa-level forest plots suggest a pattern of reduced *Lactobacillus* abundance and increased abundance of several opportunistic or anaerobe-associated taxa in infertile women. However, the strength and interpretability of these signals varied across taxa and sites, with the clearest pattern observed for vaginal *Lactobacillus*.

### 3.4 β-diversity

Sixteen studies (16/27, 59.26%) (Dai et al., 2016; Campisciano et al., 2017; Huang et al., 2019; Zhao et al., 2020, 2025; Chen et al., 2021, 2025b; Fernández et al., 2021; Ichiyama et al., 2021; Manzoor et al., 2021; Dong et al., 2023; Chopra et al., 2024; Haahr et al., 2024; Hasan et al., 2024; Su et al., 2024; Gao et al., 2025) reported β-diversity outcome, with thirteen studies (13/16, 81.25%) indicating significant differences in β-diversity between infertile women and controls (see **Supplementary Table 4**).

### 3.5 Risk of bias assessment

Twenty studies (20/27, 74.07%) achieved a NOS score of 7–8 which were indicated as high quality, seven studies (7/27, 25.93%) achieved a score of 4–6 with the lowest score being 4, indicated as moderate quality (see **Supplementary Table 5**). In the GRADE evidence profile (see **Supplementary Table 7**), two out of ten microbial taxa (Shannon index and *Prevotella*) received a rating of low-quality evidence, while the remaining microbial taxa received a rating of very-low-quality evidence.

### 3.6 Sensitivity analyses

Sensitivity analyses were conducted for outcomes with moderate heterogeneity and more than two included studies. Sequential exclusion of individual studies revealed minimal changes in SMD values and 95% CrIs for the Shannon index, Observed index, and Chao1 index. After excluding six studies individually, the 95% CrI for the Simpson index included the null value of 0, but the SMD remained greater than 0. Others have no significant changes (see **Supplementary Table 6**).

### 3.7 Meta-regression analyses

BMI showed positive associations with the Observed index (P<0.001) and ACE index (P = 0.013). However, these findings were based on very small numbers of effect sizes (k = 3 and k = 4, respectively) (see **Supplementary Table 10**).

## 4 Discussion

This systematic review and meta-analysis investigated changes in the reproductive tract microbiota associated with female infertility. All included studies were observational, and the overall certainty of evidence was low or very low. These findings should be interpreted as associations and the taxa identified here should be regarded as hypothesis-generating microbial signals requiring external validation.

The interpretation of increased α-diversity requires site-specific caution. In vaginal samples, greater Shannon, Simpson, or ACE indices may reflect reduced *Lactobacillus* dominance and a shift toward a more polymicrobial community, a pattern often described in bacterial vaginosis-related or inflammatory states (Chee et al., 2020). In contrast, the biological meaning of diversity in the cervix and endometrium is less certain. The endometrium is a low-biomass environment, and its baseline microbial diversity, contamination risk, and relationship with reproductive outcomes remain debated (Moreno et al., 2016; Toson et al., 2022). Thus, increased diversity should not be uniformly equated with “dysbiosis” across all reproductive tract sites. In this manuscript, dysbiosis is used to denote a deviation from expected site-specific microbial community structure, rather than a proven pathological mechanism.

Decreased relative abundance of *Lactobacillus*, L. crispatus, and L. iners was most interpretable in vaginal samples, where *Lactobacillus* dominance is commonly considered a feature of reproductive tract microbial stability. A possible explanation is that reduced *Lactobacillus* may weaken lactic-acid-mediated ecological restraint and allow expansion of anaerobic or opportunistic taxa (Chee et al., 2020). This explanation remains indirect, as the included studies did not experimentally assess lactic acid production, mucosal immunity, epithelial integrity, or inflammatory pathways. Moreover, *Lactobacillus* abundance is not linearly beneficial in all ART contexts, as some profiles with high L. crispatus abundance have been associated with less favorable *in vitro* fertilization (IVF) outcomes (Koedooder et al., 2019).

*Prevotella* is frequently reported in bacterial vaginosis and other inflammatory gynecological conditions and is not specific to infertility (Gao et al., 2024). Some *Prevotella* species may contribute to local inflammatory signaling through endotoxin-related pathways, but such mechanisms were not directly tested in the included studies (Aroutcheva et al., 2008). *Burkholderia* is an opportunistic and environment-associated genus; in low-biomass cervical or endometrial samples, its detection may reflect true colonization, environmental exposure, reagent contamination, or sampling contamination if negative controls are insufficient (Gao et al., 2024; Khan et al., 2024). The reduction in *Enterococcus* should also not be overinterpreted. *Enterococcus* is usually present at low abundance and may be influenced by antibiotic exposure, inter-microbial competition, or study-specific detection thresholds. Thus, these taxa should be considered non-specific microbial signals rather than infertility-specific biomarkers.

This study has several limitations. First, substantial heterogeneity and residual confounding limit the biological interpretation of the pooled estimates. The included studies differed in anatomical site, sampling and sequencing methods, bioinformatic pipelines, infertility etiology, menstrual-cycle and hormonal status, antibiotic or probiotic exposure, ART history, sexual behavior, ethnicity, and BMI. Although subgroup analyses and meta-regression were conducted, these approaches only partially addressed such variability because they relied on aggregate study-level data, were limited by incomplete reporting, and could not replace individual-level adjustment. The meta-regression findings did not identify a stable explanatory factor for most α-diversity outcomes, and the BMI-related signals for some richness indices should be regarded as exploratory. Therefore, pooling vaginal, cervical, and endometrial data should be interpreted as a broad descriptive summary rather than evidence of a uniform reproductive-tract microbial pattern. Site-specific biology, ecological bias, and unmeasured confounders remain important considerations when interpreting these associations.

Second, the taxa-level synthesis has methodological limitations because relative abundance data are compositional, bounded, often zero-inflated, and interdependent. Thus, SMD should be interpreted as descriptive contrasts rather than direct biological effect sizes. Future studies should use compositional-aware methods and absolute quantification when possible.

Third, some numerical data were extracted from published plots when raw values were unavailable. This may introduce measurement error, particularly for diversity indices and taxa-level relative abundance estimates, and may have affected the precision of pooled results.

Fourth, publication bias and selective reporting may have influenced the findings. Because only taxa reported in at least two studies were analyzed, frequently reported organisms may have been preferentially included, whereas less commonly reported but potentially relevant taxa may have been missed. Therefore, taxa-level results may reflect both biological differences and reporting practices.

Fifth, the clinical relevance of these findings remains limited. The present study did not define diagnostic thresholds, develop predictive models, or demonstrate added value beyond clinical variables, and some taxa-level effect sizes were modest. Current evidence therefore does not support microbiome-based screening, treatment, or ART decision-making based solely on these taxa.

In summary, female infertility was associated with altered reproductive tract microbiota, with the most consistent findings observed in vaginal samples and less consistent evidence from cervical and endometrial samples. Increased α-diversity, reduced *Lactobacillus*, and increased *Prevotella* or *Burkholderia* should be interpreted as associative patterns. Future research should prioritize larger-scale, high-quality prospective studies, adopt standardized sampling and sequencing protocols, and integrate multi-omics data to explore longitudinal dynamics.

## Data Availability

The original contributions presented in the study are included in the article/**Supplementary Material**. Further inquiries can be directed to the corresponding authors.
