# Supplementary material for: Distinctive reproductive tract microbial characteristics associated with infertility in women: a systematic review and meta-analysis
Source: Front Cell Infect Microbiol. 2026 Jun 18;16:1836100. doi: 10.3389/fcimb.2026.1836100 (PMC13323244; doi:10.3389/fcimb.2026.1836100)
Supplement: Supplementary file 1 [file DataSheet1.pdf]

Supplemental Online Content

Distinctive Reproductive Tract Microbial Characteristics and Potential Biomarkers in Women with Infertility: A Systematic Review and Meta-Analysis

Contents

Table S1. Search strategies 2

Figure S1. Flowchart of literature screening 4

Table S2. Reasons for the final exclusion of the probable literature. 5

Table S3. Characteristics of studies included. 10

Figure S2. Comparison results of the observed and Chao1 index between infertile women and healthy controls 12

Figure S3. Comparison results of the relative abundance of Bacillota，Enterococcus， and Megasphaera between infertile women and healthy controls 13

Figure S4. Comparison results of the relative abundance of Atopobium, Gardnerella, Bifidobacterium, and Prevotella between infertile women and healthy controls 14

Figure S5. Comparison results of the relative abundance of Delftia, Burkholderia, Ralstonia, Schlegelella, Pseudomonas, Escherichia-Shigella between infertile women and healthy controls 15

Table S4. Assessing beta diversity of infertile women compared to healthy controls. 16

Table S5. Quality assessment of included studies by Newcastle-Ottawa Scale (NOS). 17

Table S6. The results of the sensitivity analysis. 20

Table S7. GRADE evidence profile for biomarkers quantified in ＞2 studies 23

Table S8 PRISMA 2020 checklist 25

Table S9 Studies with data extracted from figures using web-based tools 28

Table S10 Meta-regression analysis for α-diversity 29

Table S1. Search strategies

Databases	Search strategies	Results	Time
MEDLINE	#1: ((((((((Infertility, Female[MeSH Terms]) OR (Sterility[Title/Abstract])) OR (Subfertility[Title/Abstract])) OR (Infertility[Title/Abstract])) OR (Fertilization in Vitro[MeSH Terms])) OR (Fertilization in Vitro[Title/Abstract])) OR (IVF[Title/Abstract])) OR (Test Tube Fertilization[Title/Abstract])) OR (Test-Tube Baby[Title/Abstract])<br>#2: ((((((((Microbiota[MeSH Terms]) OR (Microbiota[Title/Abstract])) OR (Microbiome[Title/Abstract])) OR (Microbial Community[Title/Abstract])) OR (Ecosystem[Title/Abstract])) OR (Bacteria[Title/Abstract])) OR (Flora[Title/Abstract])) OR (Microflora[Title/Abstract])) OR (Dysbiosis[Title/Abstract])<br>#3: (((((((((((Vagina[MeSH Terms]) OR (Vagina[Title/Abstract])) OR (Virginia[Title/Abstract])) OR (Vaginal[Title/Abstract])) OR (Genital tract[Title/Abstract])) OR (Reproductive tract[Title/Abstract])) OR (Urogenital[Title/Abstract])) OR (Cervical[Title/Abstract])) OR (Uterus[MeSH Terms])) OR (Uterus[Title/Abstract])) OR (Womb[Title/Abstract])) OR (Uteri[Title/Abstract])<br>#4: #2 AND #3<br>#5: #1 AND #4	845	25/12/30
WOS	#1: TS=(Infertility, Female OR Sterility OR Subfertility OR Infertility OR Fertilization in Vitro OR IVF OR Test Tube Fertilization OR Test-Tube Baby) and Preprint Citation Index (Exclude – Database) and Research Commons (Exclude – Database)<br>#2: TS=(Microbiota OR Microbiome OR Microbial Community OR Ecosystem OR Bacteria OR Flora OR Microflora OR Dysbiosis) and Preprint Citation Index (Exclude – Database) and Research Commons (Exclude – Database)<br>#3: TS=(Vagina OR Virginia OR Vaginal OR Genital tract OR Reproductive tract OR Urogenital OR Cervical OR Uterus OR Womb OR Uteri) and Preprint Citation Index (Exclude – Database) and Research Commons (Exclude – Database)<br>#4: #2 AND #3 and Preprint Citation Index (Exclude – Database) and Research Commons (Exclude – Database)<br>#5: #1 AND #4 and Preprint Citation Index (Exclude – Database) and Research Commons (Exclude – Database)	3154	25/12/30
CENTRAL	#1: MeSH descriptor: [Infertility, Female] explode all trees<br>#2: MeSH descriptor: [Fertilization in Vitro] explode all trees<br>#3: (Sterility):ti,ab,kw or (Subfertility):ti,ab,kw or (Infertility):ti,ab,kw or (Fertilization in Vitro):ti,ab,kw or (IVF):ti,ab,kw or (Test Tube Fertilization):ti,ab,kw or (Test-Tube Baby):ti,ab,kw<br>#4: MeSH descriptor: [Microbiota] explode all trees<br>#5: (Microbiota):ti,ab,kw or (Microbiome):ti,ab,kw or (Microbial Community):ti,ab,kw or (Ecosystem):ti,ab,kw or (Bacteria):ti,ab,kw or (Flora):ti,ab,kw or (Microflora):ti,ab,kw or (Dysbiosis):ti,ab,kw	85	25/12/30

Databases	Search strategies	Results	Time
	#6: MeSH descriptor: [Vagina] explode all trees<br>#7: MeSH descriptor: [Uterus] explode all trees<br>#8: (Vagina):ti,ab,kw or (Virginia):ti,ab,kw or (Vaginal):ti,ab,kw or (Genital tract):ti,ab,kw or (Reproductive tract):ti,ab,kw or (Urogenital):ti,ab,kw or (Cervical):ti,ab,kw or (Uterus):ti,ab,kw or (Womb):ti,ab,kw or (Uteri):ti,ab,kw<br>#9: #1 or #2 or #3<br>#10: #4 or #5<br>#11: #6 or #7 or #8<br>#12: #10 and #11<br>#13: #9 and #12		
EMBASE	#1: 'infertility, female':ab,ti OR 'sterility':ab,ti OR 'subfertility':ab,ti OR 'infertility':ab,ti OR 'fertilization in vitro':ab,ti OR 'ivf':ab,ti OR 'test tube fertilization':ab,ti OR 'test-tube baby':ab,ti<br>#2: 'microbiota':ab,ti OR 'microbiome':ab,ti OR 'microbial community':ab,ti OR 'ecosystem':ab,ti OR 'bacteria':ab,ti OR 'flora':ab,ti OR 'microflora':ab,ti OR 'dysbiosis':ab,ti<br>#3: 'vagina':ab,ti OR 'virginia':ab,ti OR 'vaginal':ab,ti OR 'genital tract':ab,ti OR 'reproductive tract':ab,ti OR 'urogenital':ab,ti OR 'cervical':ab,ti OR 'uterus':ab,ti OR 'womb':ab,ti OR 'uteri':ab,ti<br>#4: #2 AND #3<br>#5: #1 AND #4	1045	25/12/30

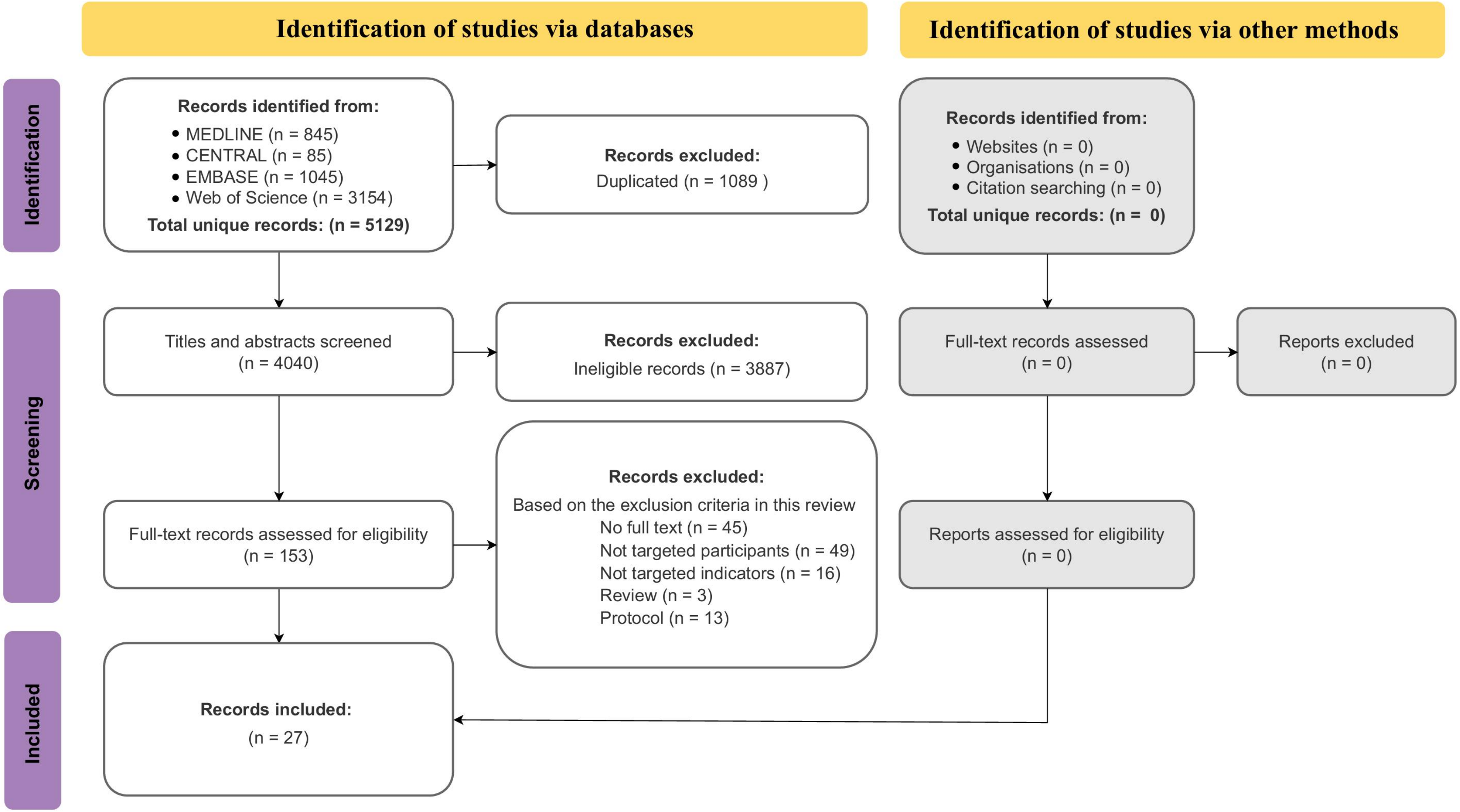

Figure S1. Flowchart of literature screening

Table S2. Reasons for the final exclusion of the probable literature.

NO.	Title	Reason
1	ASSESSING THE FEASIBILITY OF PREOPERATIVE PREVENTIVE VAGINAL CLEANSING TO PREVENT CONTAMINATION OF THE UTERINE CAVITY WITH VAGINAL MICROFLORA	No full text
2	1.\tComparison of endometrial versus vaginal microbiota in 71 infertile patients	No full text
3	101 The association between total bacterial load, the amount and proportion of lactobacillus in vaginal and endometrial samples	Not targeted indicators
4	16S rRNA long-read nanopore sequencing is feasible and reliable for endometrial microbiome analysis.	Not targeted indicators
5	16s sequencing of vaginal microbiota in patients undergoing frozen embryo transfer following IVF	No full text
6	A Double-blind, Placebo-controlled Multicenter Trial on the Effect of Clindamycin and a Live Biotherapeutic on the Reproductive Outcomes of IVF Patients With Abnormal Vaginal Microbiota	protocol
7	A microbiome based strategy for the prediction of infectious infertility	protocol
8	A More Diverse Cervical Microbiome Associates with Better Clinical Outcomes in Patients with Endometriosis: A Pilot Study.	Not targeted participants
9	A multicenter retrospective study of the investigation of detailed immunological inflammatory grading for chronic endometritis and their correlation with the endometrial microbiome	Not targeted indicators
10	A New Methodology to Assess Fallopian Tubes Microbiota and Its Impact on Female Fertility	protocol
11	A nomogram prediction model for embryo implantation outcomes based on the cervical microbiota of the infertile patients during IVF-FET	Not targeted participants
12	A pilot study using unique targeted testing of the urogenital microbiome has potential as a predictive test during IVF for implantation outcome	Not targeted participants
13	A Randomized Controlled Trial of a Nutritional Intervention for Endometriosis	protocol
14	A RANDOMIZED, DOUBLE BLIND, PLACEBO CONTROLLED TRIAL ASSESSING THE EFFECTS OF A VAGINALLY ADMINISTERED PHARMACOBIOTIC OVULE ON THE VAGINAL MICROBIOME IN WOMEN UNDERGOING CONTROLLED OVARIAN STIMULATION	No full text
15	A Randomized, Double-blind, Placebo-controlled Study to Confirm the Positive Effect of Fertibiome® (Ligilactobacillus Salivarius PS11610) on the Female Genital Tract Microbiota of Couples or Women With Fertility Disorders.	protocol
16	A Study on the Correlation Between Endometriosis and Intra-tissue Microbiota	protocol
17	Abnormal vaginal microbiota may be associated with poor reproductive outcomes: A prospective study in IVF patients	Not targeted participants
18	Adverse effect of lactobacilli-depauperate cervicovaginal microbiota on pregnancy outcomes in women undergoing frozen-thawed embryo transfer	Not targeted participants
19	Alteration of microbial communities and immune populations in patients with endometriosis	No full text
20	Alterations in vaginal microbiota and associated metabolome in women with recurrent implantation failure	Not targeted participants
21	Altered Endometrial Microbiota Profile Is Associated With Poor Endometrial Receptivity of Repeated Implantation Failure	Not targeted participants
22	Altered microbiota in the female reproductive tract as a risk factor for failure of assisted reproductive technologies	Not targeted participants
23	An Altered Endometrial Microbiome Activates Immune System and Defense Response in the Endometrium of Women with Reproductive Failure after IVF	No full text
24	An Analysis of the Digestive and Reproductive Tract Microbiota in Infertile Women with Obesity	Not targeted participants
25	Analysis of the microbiota composition in the genital tract of infertile patients with chronic endometritis or endometrial polyps.	Not targeted participants
26	Analysis of vaginal and endometrial microbiome in infertile women	review
27	Analysis of vaginal and uterine microbiota of infertile and recurrent miscarriage patients	protocol
28	Assessing the Endometrial Environment in Recurrent Pregnancy Loss and Unexplained Infertility	protocol
29	Assessing the endometrium in recurrent implantation failure (RIF) - a prospective controlled cohort study	No full text
30	Assessing the female reproductive tract microbiome: evidence of a continuous microbial landscape	No full text
31	Assessment of bacterial diversity associated with assisted reproductive technologies through next-generation sequencing	Not targeted participants

32	Assessment of the intestinal and vagina microbioma during extracorporal fertilization programs	Not targeted indicators
33	Association between torquetenovirus in vaginal secretions and infertility: An exploratory metagenomic analysis	Not targeted participants
34	Association of abnormal vaginal flora with increased cervical tumour necrosis factor--alpha and interferon--gamma levels in idiopathic infertility.	Not targeted indicators
35	Association of opportunistic bacterial pathogens with female infertility: A case–control study	Not targeted indicators
36	Association of the Cervical Microbiota With Pregnancy Outcome in a Subfertile Population Undergoing In Vitro Fertilization: A Case-Control Study	Not targeted participants
37	Association of the vaginal microbiome with prophylactic antibiotic exposure and clinical outcomes in women undergoing in vitro fertilization: a randomized controlled pilot study	No full text
38	Association of vaginal bacterial communities and reproductive outcomes with prophylactic antibiotic exposure in a subfertile population undergoing in vitro fertilization: a prospective exploratory study	Not targeted indicators
39	Associations Between the Vaginal Microbiome, Inflammatory Status and Pregnancy Outcome - a Prospective, Observational Study in Women Undergoing Frozen Embryo Transfers.	protocol
40	Associations between vaginal flora, MIP-1α, IL-17A, and clinical pregnancy rate in AIH.	Not targeted indicators
41	Bacterial Vaginosis (BV) and Vaginal Microbiome Disorders in Women Suffering from Polycystic Ovary Syndrome (PCOS)	Not targeted participants
42	Biochemical indicators and vaginal microecological abnormalities indicate the occurrence of intrauterine adhesions	Not targeted participants
43	Biological Vulnerability to Chlamydia trachomatis in Adolescents and Young Women: the Complex Intersection of Cervicovaginal Microbiome, Cervical Maturation, and Mucosal Immunity	No full text
44	Changes in the predominant human Lactobacillus flora during in vitro fertilisation	Not targeted participants
45	Characterisation of the human uterine microbiome in non-pregnant women through deep sequencing of the V1-2 region of the 16S rRNA gene	Not targeted participants
46	Characterising the vaginal microbiome using state-of-the-art metagenomics among women in the general British population to inform sexual and reproduct	No full text
47	CHARACTERISTICS OF THE VAGINAL MICROBIOTA, CERVICAL AND UTERINE FLORA IN WOMEN WITH THE PAST HISTORY OF SEXUALLY TRANSMITTED INFECTIONS	Not targeted indicators
48	Characterization of Microbiota in Endometrial Fluid and Vaginal Secretions in Infertile Women with Repeated Implantation Failure.	Not targeted participants
49	Characterization of the vaginal microbiome of Brazilian women of reproductive age	No full text
50	Characterization of the vaginal microbiota in infertile women with repeated implantation failure	Not targeted participants
51	Characterization of Vaginal microbiota and reproductive outcomes associated with assisted reproductive technologies through next-generation sequencing, from Indian population	No full text
52	Characterization of vaginal microbiota during IVF fresh embryo transfer (IVF-ET) and in early pregnancy	No full text
53	CHARACTERIZATION OF VAGINAL SECRETION MICROBIOTA IN INFERTILE WOMEN WITH CHRONIC ENDOMETRITIS	No full text
54	CHARACTERIZING REPRODUCTIVE TRACT MICROBIOME THROUGH SHOTGUN METAGENOMIC SEQUENCING AT TIME OF IVF, A PILOT STUDY	No full text
55	Characterizing the endometrial microbiome by analyzing the ultra-low bacteria from embryo transfer catheter tips in IVF cycles: Next generation sequencing (NGS) analysis of the 16S ribosomal gene	Not targeted indicators
56	Chronic endometritis and the endometrial microbiota: implications for reproductive success in patients with recurrent implantation failure	Not targeted participants
57	Clinical Applicability of Microbiota Sampling in a Subfertile Population: Urine versus Vagina.	Not targeted participants
58	Combining deep shotgun metagenomic sequencing and culturomics for in-depth characterization of a potential endometrial microbiome	No full text

59	Comparative Analysis of Lower Genital Tract Microbiome Between PCOS and Healthy Women	Not targeted participants
60	Comparative study on the vaginal flora and incidence of asymptomatic vaginosis among healthy women and in women with infertility problems of reproductive age	Not targeted indicators
61	COMPARING the UTERINE MICROBIOME in RECURRENT PREGNANCY LOSS to PAROUS FERTILE CONTROLS	No full text
62	Comparison of microbial abundance and diversity in uterine and peritoneal fluid in infertile patients with or without endometriosis	Not targeted participants
63	Comparison of microbial profiles and viral status along the vagina-cervix-endometrium continuum of infertile patients	Not targeted participants
64	Comparison of the Urinary and Vaginal Microbiota in Women with Different Subfertility Diagnoses: Escherichia Coli Specific for Male Factor	No full text
65	Comparison of vaginal microbiota and inflammation between people with unexplained vs. male factor infertility	No full text
66	Composition of the endometrial microbiome is associated to reproductive outcomes in IVF patients	No full text
67	Comprehensive analysis of vaginal microbiota in Chinese women with genital tuberculosis: implications for diagnosis and treatment	Not targeted participants
68	Correlation between endometriosis, infertility and bacterial population of the uterus of women with endometriosis and infertility	Not targeted indicators
69	Culturomics-generated vaginal and endometrial microbiome profiles in subfertile patients	No full text
70	Deep Grouping Analysis of the Altered Cervical Canal Microbiota in Intrauterine Adhesion Patients	Not targeted participants
71	Differences in endometrial microbial composition correlate with implantation failure after IVF embryo transfer	No full text
72	Differences in microbial profile of endometrial fluid and tissue samples in women with in vitro fertilization failure are driven by Lactobacillus abundance.	Not targeted participants
73	Differential characteristics of vaginal versus endometrial microbiota in IVF patients	Not targeted participants
74	Differential composition of vaginal microbiome, but not of seminal microbiome, is associated with successful intrauterine insemination in couples with idiopathic infertility: A prospective observational study	Not targeted participants
75	Discrepancies between vaginal and endometrial microbiomes in IVF patients: implications for reproductive success	No full text
76	Distinct Gastrointestinal and Reproductive Microbial Patterns in Female Holobiont of Infertility.	Not targeted participants
77	Distinct vaginal microbiome profile was observed in Japanese IVF patients	No full text
78	Distribution of pathogenic microbes of reproductive tract in patients with tubal infertility	No full text
79	Does the uterine microbiota affect the reproductive outcomes in women with recurrent implantation failures?	Not targeted participants
80	Effect of Comprehensive Individualised Interventions on the Clinical Outcomes of Patients With Recurrent Implantation Failure: A Single-Centre Retrospective Cohort Study.	Not targeted indicators
81	Endometrial factors in the implantation failure spectrum: protocol of a MUltidisciplinary observational cohort study in women with Repeated Implantation failure and recurrent Miscarriage (MURIM Study).	protocol
82	Endometrial Flora Sampling in Women Undergoing IVF/PGD Treatments	protocol
83	Endometrial microbiome during early pregnancy among women with and without chronic endometritis: a pilot study.	Not targeted participants

84	Endometrial microbiome in women with and without a history of repeated failures of assisted reproductive technology: What are norm and pathology?	No full text
85	Endometrial microbiota composition is associated with reproductive outcome in infertile patients	Not targeted participants
86	Evaluating Historical Paradigms of Sterility in Perinatal Microbiology and Ramifications for Pregnancy Outcomes	No full text
87	Exploring the relationship between the vaginal microbiota and vaginal symptoms	No full text
88	Features of inter-microbial relations in the infertile women's vagina microbiota	Not targeted participants
89	Genital tract dysbiosis in infertile women with a history of repeated implantation failure and pilot study for reproductive outcomes following oral enteric coating lactoferrin supplementation.	Not targeted participants
90	GUT AND VAGINAL MICROBIOTA IN FEMALE WITH REPRODUCTIVE DISORDERS ASSOCIATED WITH GASTROINTESTINAL DISTURBANCE	No full text
91	High-throughput Sequencing Analysis of Reproductive Tract Microbiota in Patients with Moderate and Severe Intrauterine Adhesions	Not targeted participants
92	Identification of vaginal microbiome associated with IVF pregnancy	Not targeted participants
93	Impact of Lactobacillus in the uterine microbiota on in vitro fertilization outcomes.	Not targeted participants
94	Implantation failure is not associated with seven different vaginal bacterial community-state types; however, a distinct signature, including Ureaplasma parvum, is strongly linked to treatment failure	No full text
95	Interaction of male and female genital microbiota in infertile couples undergoing assisted reproductive technology	No full text
96	INTERPLAY BETWEEN ENDOMETRIAL MICROBIOTA AND ANTIMICROBIAL PEPTIDES IN WOMEN WITH DIFFERENT INFERTILITY FORMS	Not targeted indicators
97	Longitudinal Study of Vaginal Microbiota in Pregnant Women Following in Vitro Fertilization	Not targeted participants
98	Microbiota in Endometrial Fluid and Vaginal Secretions in Infertile Women with a History of Repeated Implantation Failure	No full text
99	Microflora in female reproductive tract of women with tubal infertility	Not targeted indicators
100	NEW RELATION BETWEEN DYSBIOSIS OF THE VAGINAL AND ENDOMETRIAL MICROBIOTA AND RIF FOUND	No full text
101	Patients with Repeated Implantation Failure do not have a higher prevalence of an unfavourable vaginal microbiome	No full text
102	pattern of vaginal/endometrial microbiome as a predictor for outcome of in vitro fertilization (IVF) in patients with or without repetitive implantation failure:a pilot study	No full text
103	Personalized probiotics and food supplementation according to microbiome miRNA test improves the conception rate in multiple IVF failure patients	No full text
104	Potential biomarkers of infertility associated with microbiome imbalances	Not targeted indicators
105	Refining Unfavorable Vaginal Microbial Community in Infertile Women Subjected to Precision Probiotic Intervention: An Exploratory Single-Arm, Prospective, Open-Label, Interventional Study.	Not targeted participants
106	RUBIC (ReproUnion Biobank and Infertility Cohort): A binational clinical foundation to study risk factors, life course, and treatment of infertility and infertility-related morbidity.	protocol
107	Structural Variations of Vaginal and Endometrial Microbiota: Hints on Female Infertility	Not targeted participants
108	Study on the correlation among dysbacteriosis, imbalance of cytokine and the formation of intrauterine adhesion	Not targeted participants
109	Study on the effect of vaginal microbiota on embryo implantation	Not targeted participants

110	Systematic Comparison of Bacterial Colonization of Endometrial Tissue and Fluid Samples in Recurrent Miscarriage Patients: Implications for Future Endometrial Microbiome Studies.	Not targeted participants
111	Testing on bacterial vaginosis in a subfertile population and time to pregnancy: a prospective cohort study	Not targeted participants
112	The association of lower genital infection with secondary infertility and antimicrobial sus-ceptibility analysis of microflora	Not targeted participants
113	The cervical microbiome in female infectious infertility	No full text
114	The effects of intravaginal estrogen and/or probiotic interventions on the vaginal microbiome: Reducing STI susceptibility in high-risk populations	No full text
115	The impact of the urogenital microbiome on infertility	No full text
116	The microbial community of the female urogenital tract in the context of sexually transmitted infections and infertility	No full text
117	The Microbiome of Infertile Couples and Its Effect on Their Reproductive Outcomes	protocol
118	The relationship between dysbiosis of vaginal microbiome and proinflammatory cytokine levels in vaginal fluid in recurrent implantation failure patients	No full text
119	The role of gut and genital microbiota and the estrobolome in endometriosis, infertility and chronic pelvic pain	review
120	The stability of lower genital tract (LGT) microbiota correlates with reproductive system function and in vitro fertilization and frozen embryo transfer outcomes in women with polycystic ovarian syndrome	Not targeted participants
121	The vaginal microbiome in the first trimester of pregnancy is different in spontaneous versus IVF gestation	No full text
122	To understand the reproductive tract microbiome associated with infertility through metagenomics analysis	review
123	Use of a Vaginal Probiotic Suppository and Antibiotics in the Treatment of endometrial microbiota	Not targeted participants
124	Uterine Fluid microbiome composition in fertile controls and infertile women	No full text
125	Vaginal and endometrial microbiota: is there any correlation	No full text
126	Vaginal microbiota are associated with in vitro fertilization during female infertility.	Not targeted participants

Table S3. Characteristics of studies included.

ID	Country	Disease	Control	Samplesize (Infertility/Control)	Methods	Age（mean±sd）	BMI	Indicators
Wee 2017[abc]	Australia	Infertility or IVF	Parous women	31(15/16)	16S rRNA V1-V3	40.26 (mean)	NA	Relative abundance, α diversity
Chen 2021[a]	China	Infertility	Healthy women	21(14/7)	16S rRNA V3-V4	33.86±4.01	NA	Relative abundance, Observed species, Shannon, Simpson, Chao1, ACE, Good's coverage, β diversity
Hasan 2024[a]	Bangladesh	Infertility	Parous women	10(5/5)	16S rRNA V3-V4, metagenome	NA	NA	Observed species, Shannon, Simpson, Chao1, ACE, Jackknife, Phylogenetic diversity, β diversity
Kyono 2018[ac]	Japan	Infertility or IVF	Parous women	109(102/7)	16S rRNA V4	36.17±4.51	20.51±2.92	Relative abundance
Ichiyama 2021[ac]	Japan	IVF	Healthy women	166(145/21)	16S rRNA V4	37.5±4.66	21.11±2.78	Relative abundance, Shannon, Chao1, β diversity
Fernández 2021[ab]	Spain	IVF	Parous women	37(23/14)	16S rRNA V3-V4	38.67±1.53	NA	Relative abundance, Shannon, Simpson, β diversity
Manzoor 2022[#]	Pakistan	Infertility	Parous women	30(16/14)	16S rRNA V4	28.25±5.47	NA	Relative abundance, Observed species, Shannon, Faith PD, Evennnes, β diversity
Zhao 2020[a]	China	IVF	Healthy women	80(30/50)	16S rRNA	30.72±7.27	NA	Relative abundance, Chao1, β diversity
Sun 2021[ac]	China	Infertility	Healthy women	30(15/15)	16S rRNA V1-V3	NA	NA	Relative abundance
Huang 2019[a]	China	Infertility	Parous women	30(15/15)	16S rRNA V3-V4	30.93±5.27	22.91±3.63	Relative abundance, Shannon, Simpson, Chao1, ACE, β diversity
Chen 2025_1[a]	China	Infertility	Parous women	22(15/7)	16S rRNA V3-V4	NA	NA	Relative abundance
Patel 2022[a]	India	IVF	Healthy women	24(16/8)	16S rRNA V2-V3	30.96±4.78	23.74±3.83	Relative abundance
Zhao 2025[a]	China	IVF	Parous women	114(77/37)	16S rRNA	31.41±4.95	22.22±1.82	Relative abundance, Shannon, Simpson, β diversity
Moreno 2016[ac]	Spain	IVF	Parous women	55(35/22)	16S rRNA V3-V5	NA	NA	Relative abundance, Shannon, β diversity
Mohammadi 2025[abc]	Iran	IVF	Healthy women	50(40/10)	16S rRNA	NA	NA	Relative abundance
Gao 2025[c]	China	IVF	Healthy women	36(17/19)	16S rRNA V3-V4	32.66±5.17	22.22±2.86	Relative abundance, Shannon, Chao1, β diversity
Vladislavovna 2020[c]	Russia	IVF	Healthy women	42(22/20)	16S rRNA V3-V4	31.61±4.59	22.02±2.08	Relative abundance
Graspeuntner 2018[b]	Germany	Infertility	Parous women	136(47/89)	16S rRNA V3-V4	NA	NA	Relative abundance, Simpson

Aldhalimi 2025[a]	Iraqi	Infertility	Parous women	310(210/100)	16S rRNA	30.51±5.08	26.71±3.58	Shannon, Simpson
Chopra 2024[a]	India	Infertility	Parous women	80(40/40)	16S rRNA V3-V4	25.72±5.39	24.3±5.04	Relative abundance, Observed species, Shannon, Simpson, Chao1, ACE, Fisher, β diversity
Dai 2016[a]	China	Infertility	Parous women	80(40/40)	16S rRNA V4-V5	30.73±5.64	NA	Relative abundance, Shannon, β diversity
Campisciano 2017[a]	Italy	Infertility	Parous women	96(27/69)	16S rRNA V3	35.53	NA	Relative abundance, Chao1, Simpson, β diversity
Haahr 2024[abc]	Denmark, Spain	IVF	Healthy women	53(27/26)	16S rRNA V3-V4	30.08±4	NA	Relative abundance, β diversity
Chen 2025_2[a]	China	IVF	Parous women	294(194/100)	16S rRNA V3-V4	31.9±4.71	NA	Relative abundance, Observed species, Shannon, Chao1, ACE, β diversity
Dong 2023[ab]	China	IVF	Parous women	32(22/10)	16S rRNA	32.15±4.7	22.82±2.95	Relative abundance, Shannon, Chao1, ACE, Simpson, Observed species, β diversity
Barinova 2021[c]	Russia	IVF	Parous women	35(20/15)	16S rRNA V3-V4	32.74±4.96	22.73±3.82	Relative abundance
Su 2024[abc]	China	IVF	Parous women	171(154/17)	16S rRNA V3-V4	NA	NA	Relative abundance, Faith, β diversity

Table S3 presents the characteristics of the included studies, including ID (composed of the author and publication year), sample source (a, b, c respectively indicates samples from the vagina, cervix, and endometrium), country where the study was conducted, sample size, sequencing technology, age, body mass index (BMI), and reported indicators. In the Disease column, IVF indicates that the infertile female patients in the corresponding study are undergoing or will undergo assisted reproductive technology via in vitro fertilization (IVF); otherwise, it is marked as Infertility. In the Control column, Parous women indicates that the inclusion criteria for the control group in the corresponding study include a fertility history; otherwise, it is marked as healthy women, indicating no fertility history but no obvious factors impeding fertility upon examination. BMI, body mass index; IVF, in vitro fertilization; NA, not mentioned.

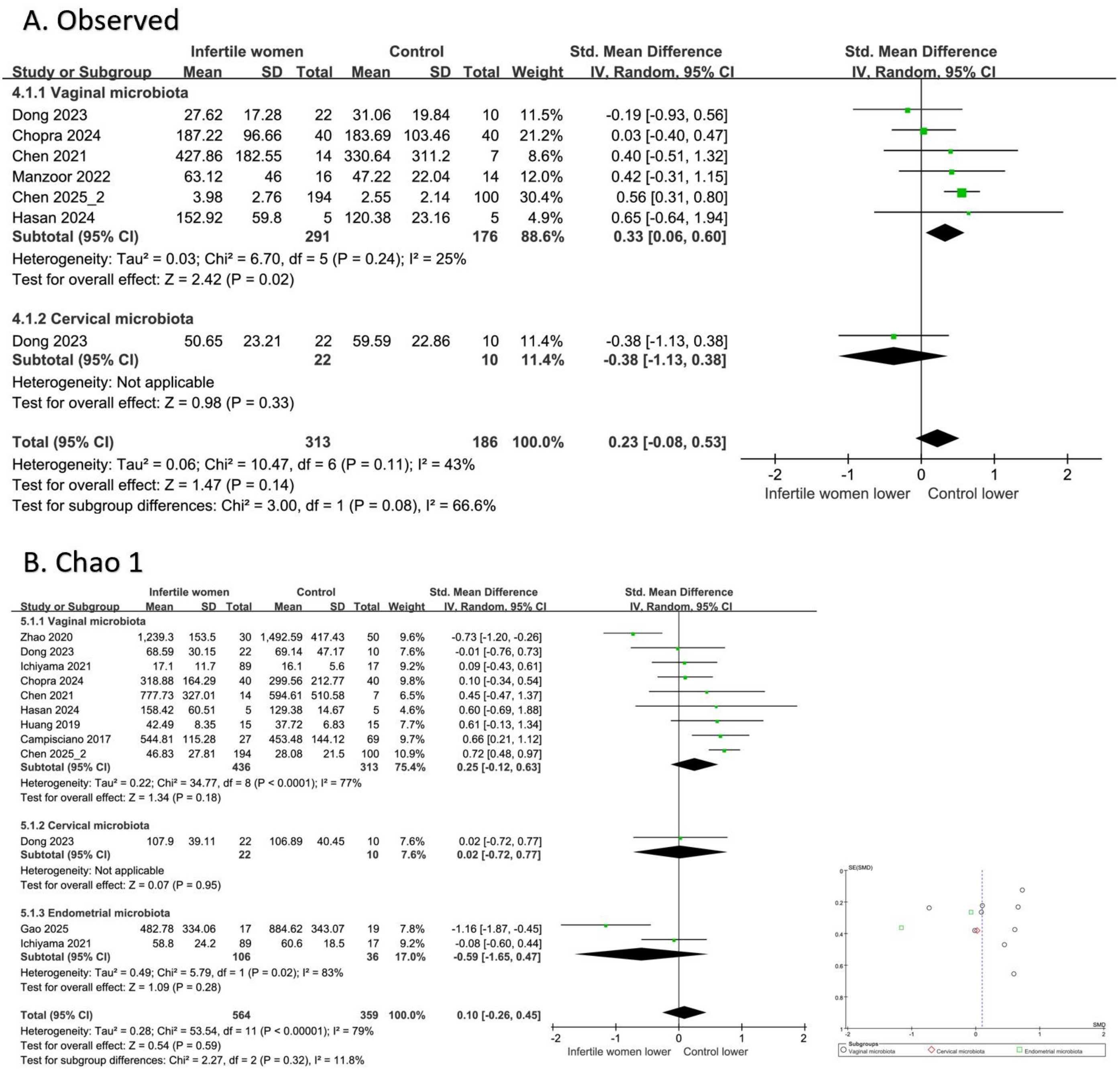

Figure S2. Comparison results of the observed and Chao1 index between infertile women and healthy controls

Figure S2 displays the forest plot (A) for the Observed index and the forest plot and funnel plot (B) for the Chao1 index, comparing the reproductive tract microbiota between infertile women and healthy controls. A random-effects model was used, with results presented as standardized mean differences (SMD) and 95% confidence intervals (CI). Subgroup analyses were performed based on whether the samples were derived from the vagina, cervix, or endometrium.

Phylum: Bacillota

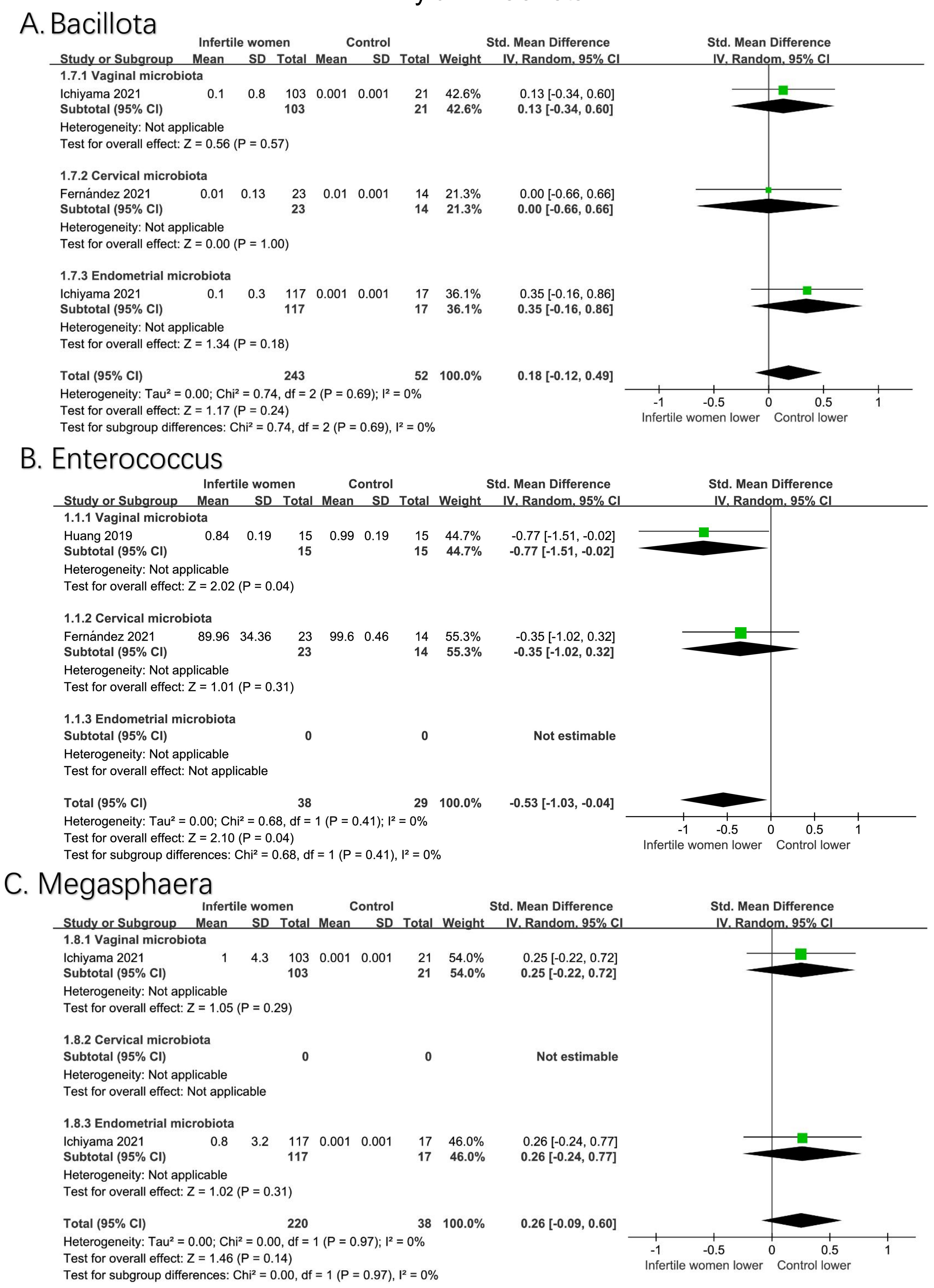

Figure S3. Comparison results of the relative abundance of Bacillota, Enterococcus, and Megasphaera between infertile women and healthy controls

Figure S3 displays the forest plots comparing the relative abundances of Bacillota (A), Enterococcus (B), and Megasphaera (C) in the reproductive tract microbiota between infertile women and healthy controls. A random-effects model was used, with results presented as standardized mean differences (SMD) and 95% confidence intervals (CI). Subgroup analyses were performed based on whether the samples were derived from the vagina, cervix, or endometrium.

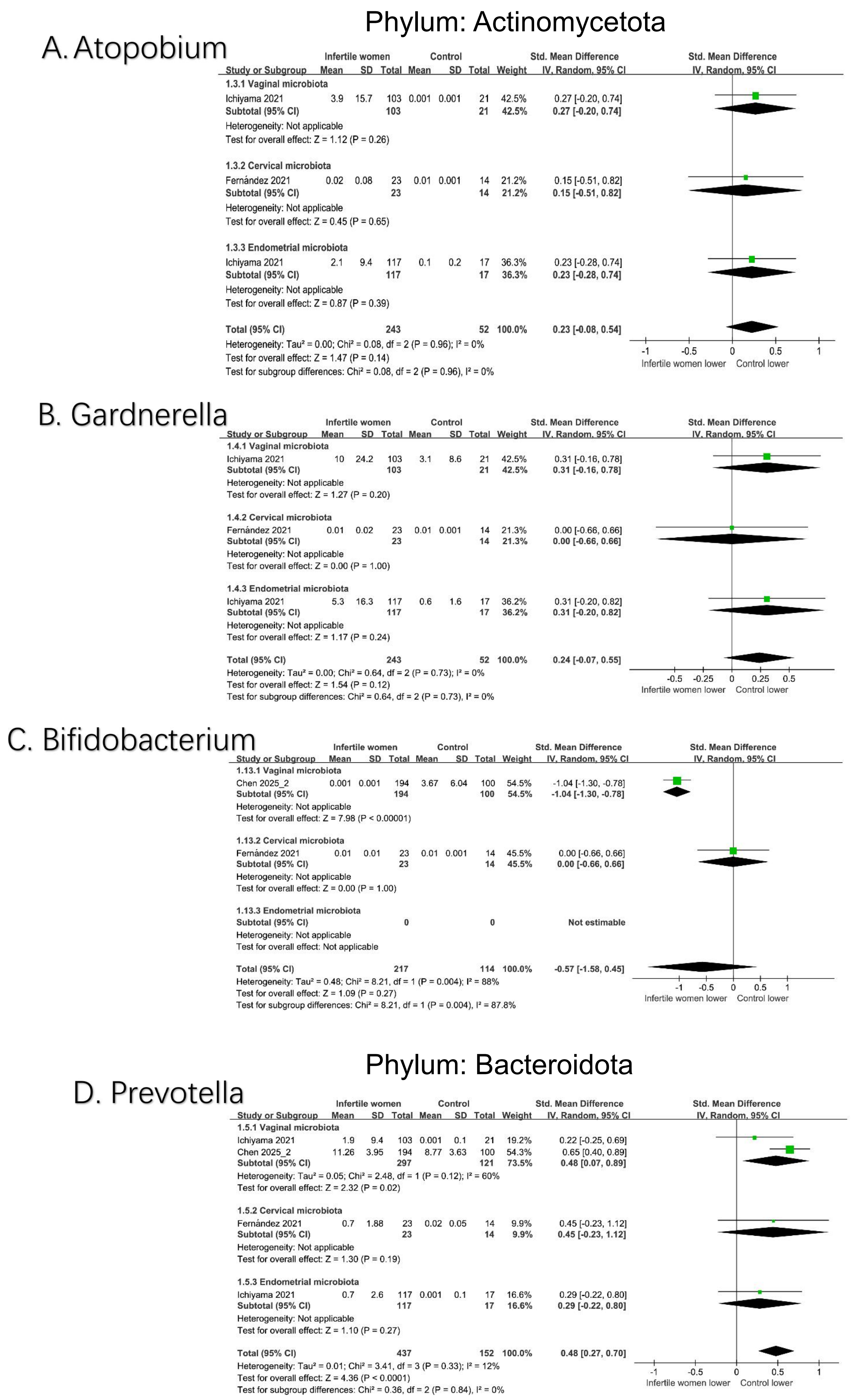

Figure S4. Comparison results of the relative abundance of Atopobium, Gardnerella, Bifidobacterium, and Prevotella between infertile women and healthy controls

Figure S4 displays the forest plots comparing the relative abundances of Atopobium (A), Gardnerella (B), Bifidobacterium (C), and Prevotella (D) in the reproductive tract microbiota between infertile women and healthy controls. A random-effects model was used, with results presented as standardized mean differences (SMD) and 95% confidence intervals (CI). Subgroup analyses were performed based on whether the samples were derived from the vagina, cervix, or endometrium.

Phylum: Pseudomonadota

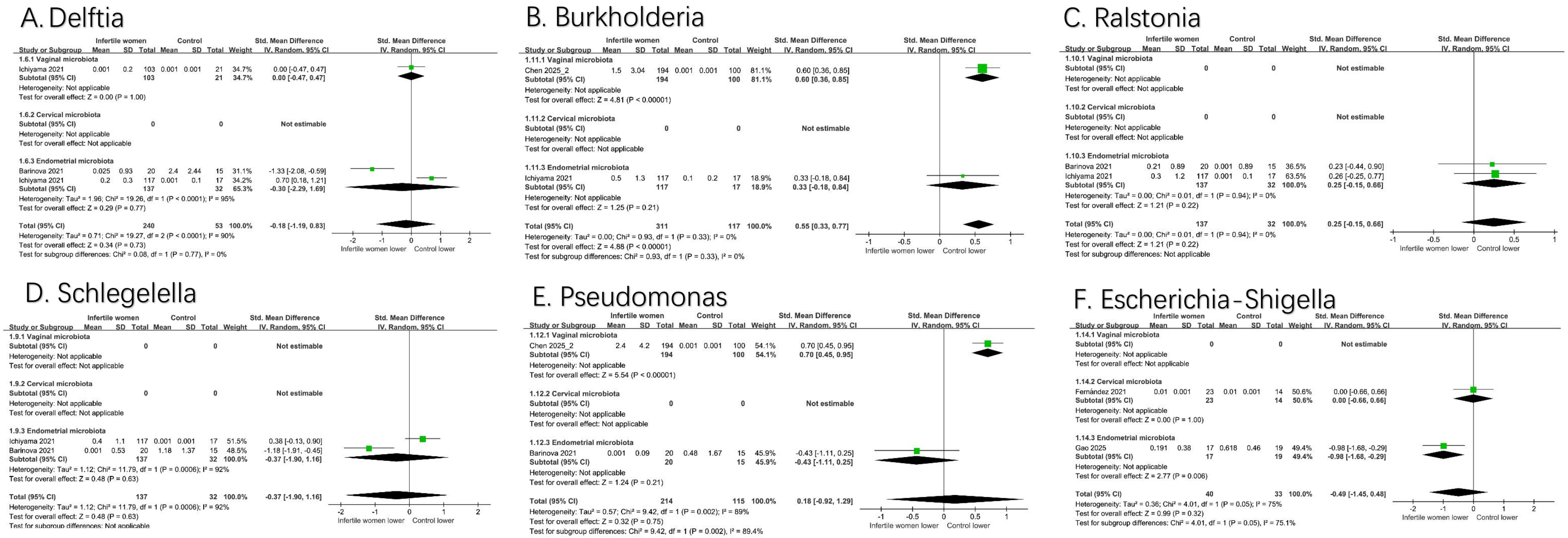

Figure S5. Comparison results of the relative abundance of Delftia, Burkholderia, Ralstonia, Schlegelella, Pseudomonas, Escherichia-Shigella between infertile women and healthy controls

Figure S5 displays the forest plots comparing the relative abundances of Delftia (A), Burkholderia (B), Ralstonia (C), Schlegelella (D), Pseudomonas (E), and Escherichia-Shigella (F) in the reproductive tract microbiota between infertile women and healthy controls. A random-effects model was used, with results presented as standardized mean differences (SMD) and 95% confidence intervals (CI). Subgroup analyses were performed based on whether the samples were derived from the vagina, cervix, or endometrium.

Table S4. Assessing beta diversity of infertile women compared to healthy controls.

ID	Metric	Finding
Chen 2021	Bray–Curtis<br>Unweighted UniFrac<br>Weighted UniFrac	No significant different
Hasan 2024	Unweighted UniFrac	No significant different
Ichiyama 2021	Weighted UniFrac	Significant different
Fernández 2021	Bray–Curtis<br>Jaccard	Significant different
Manzoor 2022	Unweighted UniFrac<br>Weighted UniFrac<br>Bray–Curtis<br>Jaccard	Significant different
Zhao 2020	Unweighted UniFrac	Significant different
Huang 2019	/	Significant different
Zhao 2025	/	Significant different
Gao 2025	Bray–Curtis	Significant different
Chopra 2024	Unweighted UniFrac	Significant different
Dai 2016	Unweighted UniFrac<br>Weighted UniFrac	No significant different
Campisciano 2017	Unweighted UniFrac<br>Weighted UniFrac	Significant different
Haahr 2024	Bray–Curtis	No significant different
Chen 2025	/	Significant different
Dong 2023	Bray–Curtis	Significant different
Su 2024	Bray–Curtis	Significant different

Table S5. Quality assessment of included studies by Newcastle-Ottawa Scale (NOS).

Study ID	Is the case definition adequate*	Representativeness of the cases*	Selection of Controls*	Definition of Controls*	Comparability of cases and controls on the basis of the design or analysis**	Ascertainment of exposure*	Same method of ascertainment for cases and controls *	Non-Response rate*	Quality score
Wee 2017	*	NA	*	*	**	NA	*	*	7
Chen 2021	NA	*	*	*	**	NA	*	*	7
Hasan 2024	NA	NA	*	*	NA	NA	*	*	4
Kyono 2018	NA	*	*	*	**	NA	*	*	7
Ichiyama 2021	*	*	*	*	**	NA	*	*	8
Fernández 2021	*	*	*	*	**	NA	*	*	8
Manzoor 2022	*	NA	*	*	**	NA	*	*	7
Zhao 2020	*	*	*	*	**	NA	*	*	8
Sun 2021	NA	NA	*	*	NA	NA	*	*	4
Huang 2019	*	*	*	*	**	NA	*	*	8
Chen 2025_1	NA	*	*	*	NA	NA	*	*	5
Patel 2022	*	*	*	*	**	NA	*	*	8
Zhao 2025	*	*	*	*	**	NA	*	*	8
Moreno 2016	*	NA	*	*	NA	*	*	*	6
Mohammadi 2025	*	NA	*	*	NA	NA	*	*	5
Gao 2025	*	*	*	*	**	NA	*	*	8
Vladislavovna 2020	*	*	*	*	**	NA	*	*	8

Graspeuntner 2018	NA	*	*	*	NA	NA	*	*	5
Aldhalimi 2025	*	*	*	*	**	NA	*	*	8
Chopra 2024	NA	*	*	*	**	NA	*	*	7
Dai 2016	*	*	*	*	**	NA	*	*	8
Campisciano 2017	NA	*	*	*	**	NA	*	*	7
Haahr 2024	*	NA	*	*	**	NA	*	*	7
Chen 2025_2	*	*	*	*	**	NA	*	*	8
Dong 2023	*	NA	*	*	**	NA	*	*	7
Barinova 2021	*	NA	*	*	**	NA	*	*	7
Su 2024	*	NA	*	*	NA	NA	*	*	5

* Means get 1 point, ** means get 2 points, / means not available, and NA means did not get the point.

Rating items	Definition
1. Is the case definition adequate	Clearly define infertility due to female factors as the inability to achieve pregnancy after 12 months or more of regular unprotected sexual intercourse to score 1 point; otherwise, score 0 points.
2. Representativeness of the cases	1 point is awarded if it is clearly stated in the article, that all eligible subjects are included over a defined period of time, or in a defined catchment area, and thus are a representative sample of the population of patients from which they are recruited.
3. Selection of Controls	1 point will be awarded if it is stated explicit that controls are recruited from the community, hospital staff or similar.
4. Definition of Controls	The control group with a history of natural conception and childbirth, or no factors hindering conception upon examination, scores 1 point; otherwise, scores 0 points.

5. Comparability of cases and controls on the basis of the design or analysis	2 stars are awarded if controls and patients are matched on age and sex and/or if these are adjusted for in the analysis. Statements of no significant differences between groups are not sufficient. If only sex or age is matched and/or adjusted for, 1 star is awarded.
6. Ascertainment of exposure	1 point will be awarded if it is specified that the laboratory staff responsible for the biomarker analysis was blinded to the case-control status of the samples.
7. Same method of ascertainment for cases and controls	1 point will be awarded if the exact same assay, quantification method and statistical analysis are used for both patients and controls.
8. Non-Response rate	This item is of no relevance and will therefore not be assessed. The maximum number of points is thus 8.

Table S6. The results of the sensitivity analysis.

1 Shannon

	Infertility sample size	Control sample size	SMD [95% CI]	I^2
Total	908	467	0.27 [0.10, 0.44]	40%
Vagina				
Omitting Chen 2021	894	460	0.26 [0.09, 0.44]	44%
Omitting Hasan 2024	903	462	0.28 [0.11, 0.45]	42%
Omitting Ichiyama 2021	819	450	0.29 [0.11, 0.47]	42%
Omitting Manzoor 2022	892	453	0.29 [0.12, 0.46]	39%
Omitting Huang 2019	893	452	0.24 [0.09, 0.38]	21%
Omitting Zhao 2025	831	430	0.28 [0.10, 0.46]	44%
Omitting Aldhalimi 2025	698	367	0.25 [0.06, 0.44]	41%
Omitting Chopra 2024	868	427	0.25 [0.07, 0.43]	41%
Omitting Dai 2016	868	427	0.28 [0.10, 0.46]	44%
Omitting Chen 2025_2	714	367	0.28 [0.08, 0.47]	44%
Omitting Dong 2023	886	457	0.27 [0.10, 0.45]	44%
Cervix				
Omitting Fernández 2021	885	453	0.24 [0.08, 0.41]	37%
Omitting Dong 2023	886	457	0.25 [0.08, 0.43]	42%
Endometrium				
Omitting Gao 2025	891	448	0.26 [0.09, 0.44]	44%
Omitting Ichiyama 2021	819	450	0.29 [0.11, 0.47]	42%
Omitting Moreno 2016	873	445	0.31 [0.15, 0.46]	29%

2 Simpson

	Infertility sample size	Control sample size	SMD [95% CI]	I^2
Total	502	396	0.24 [0.01, 0.47]	52%
Vagina				
Omitting Huang 2019	487	381	0.30 [0.08, 0.52]	44%
Omitting Dong 2023	480	386	0.27 [0.03, 0.51]	54%
Omitting Campisciano 2017	475	327	0.28 [0.03, 0.52]	51%
Omitting Zhao 2025	425	359	0.24 [-0.03, 0.50]	57%
Omitting Hasan 2024	497	391	0.24 [0.00, 0.48]	57%
Omitting Aldhalimi 2025	292	296	0.20 [-0.08, 0.48]	55%
Omitting Chen 2021	488	389	0.22 [-0.02, 0.47]	57%
Omitting Chopra 2024	462	356	0.19 [-0.06, 0.43]	50%
Cervix				
Omitting Fernández 2021	479	382	0.20 [-0.04, 0.43]	50%
Omitting Graspeuntner 2018	455	307	0.20 [-0.06, 0.47]	55%
Omitting Dong 2023	480	386	0.31 [0.10, 0.51]	40%

3 Observed

	Infertility sample size	Control sample size	SMD [95% CI]	I^2
Total	313	186	0.23 [-0.08, 0.53]	43%
Vagina				
Omitting Dong 2023	291	176	0.29 [-0.03, 0.60]	40%
Omitting Chopra 2024	273	146	0.28 [-0.07, 0.63]	38%
Omitting Chen 2021	299	179	0.20 [-0.14, 0.55]	52%
Omitting Manzoor 2022	297	172	0.19 [-0.17, 0.55]	52%

Omitting Chen 2025_2	119	86	0.07 [-0.22, 0.35]	0%
Omitting Hasan 2024	308	181	0.20 [-0.13, 0.53]	51%
Cervical				
Omitting Dong 2023	291	176	0.33 [0.06, 0.60]	25%

4 Chao 1

	Infertility sample size	Control sample size	SMD [95% CI]	I^2
Total	564	359	0.10 [-0.26, 0.45]	79%
Vagina				
Omitting Zhao 2020	534	309	0.19 [-0.13, 0.51]	71%
Omitting Dong 2023	542	349	0.11 [-0.27, 0.48]	81%
Omitting Ichiyama 2021	475	342	0.10 [-0.29, 0.48]	81%
Omitting Chopra 2024	524	319	0.10 [-0.30, 0.49]	81%
Omitting Chen 2021	550	352	0.07 [-0.30, 0.44]	81%
Omitting Hasan 2024	559	354	0.07 [-0.29, 0.44]	81%
Omitting Huang 2019	549	344	0.05 [-0.32, 0.43]	81%
Omitting Campisciano 2017	537	290	0.04 [-0.35, 0.42]	80%
Omitting Chen 2025_2	370	259	0.02 [-0.32, 0.35]	69%
Cervical				
Omitting Dong 2023	542	349	0.10 [-0.27, 0.48]	81%
Endometrium				
Omitting Ichiyama 2021	475	342	0.11 [-0.27, 0.50]	81%
Omitting Gao 2025	547	340	0.20 [-0.12, 0.52]	74%

Table S7. GRADE evidence profile for biomarkers quantified in ＞2 studies

**Quality assessment Biomarker**						
**Biomarker**	**study limitations**	**Inconsistency**	**Indirectness**	**Imprecision**	**Publication bias**	**Overall quality**
**Shannon**	No limitations (+1)	Serious inconsistency (-1)	No serious indirectness	No serious imprecision	Undetected	⊕⊕○○ **Low**
**Simpson**	No serious limitations	Very serious inconsistency (-2)	No serious indirectness	No serious imprecision	Undetected	○○○○ **Very Low**
**ACE**	No serious limitations	Serious inconsistency (-1)	No serious indirectness	Serious imprecision (-1)	Detected (-1)	○○○○ **Very Low**
**Observed**	No serious limitations	Very serious inconsistency (-2)	No serious indirectness	Serious imprecision (-1)	Undetected	○○○○ **Very Low**
**Chao1**	No limitations (+1)	Very serious inconsistency (-2)	No serious indirectness	No serious imprecision	Detected (-1)	○○○○ **Very Low**
**Bacillota**	No limitations (+1)	No serious inconsistency	No serious indirectness	Very serious imprecision (-2)	Undetected	○○○○ **Very Low**
**Atopobium**	No limitations (+1)	No serious inconsistency	No serious indirectness	Very serious imprecision (-2)	Undetected	⊕○○○ **Very Low**

**Gardnerella**	No limitations (+1)	No serious inconsistency	No serious indirectness	Very serious imprecision (-2)	Undetected	⊕○○○ **Very Low**
**Prevotella**	No limitations (+1)	No serious inconsistency	No serious indirectness	Serious imprecision (-1)	Undetected	⊕⊕○○ **Low**
**Delftia**	No limitations (+1)	No serious inconsistency	No serious indirectness	Very serious imprecision (-2)	Undetected	⊕○○○ **Very Low**

Note. We chose the starting point "Low quality" for all analyses, and perform downgrade and upgrade based on the following items.

Study limitations: based on the mean Newcastle-Ottawa criteria for case-control studies (Supplementary Table S5) total scores for the included studies; rating +1 when > 7, rating -1 when <4, and -2 when <2.

Inconsistency: based on point estimate variation, CI overlap, I^2 and p-value for I^2; rating -1 if $I^2 > 20\%$ or $p < 0.05$ and -2 if $I^2 > 40\%$ or $p < 0.01$.

Indirectness: based on differences in patients, interventions, outcomes and head-to-head comparison. As this is addressed in inclusion and exclusion criteria all analyses are rated as "no serious indirectness".

Imprecision: based on sample size requirements for effect sizes of SMD = 0.4 and 0.2; rating -1 if the total number of cases or total number of controls were < 200 and -2 if < 100.

Publication bias: assessed with funnel plots for biomarkers with > 10 studies included.

Table S8 PRISMA 2020 checklist

Section and Topic	Item #	Checklist item	Location where item is reported
**TITLE**			
Title	1	Identify the report as a systematic review.	Title
**ABSTRACT**			
Abstract	2	See the PRISMA 2020 for Abstracts checklist.	Abstract
**INTRODUCTION**			
Rationale	3	Describe the rationale for the review in the context of existing knowledge.	The 1-2 paragraph in introduction
Objectives	4	Provide an explicit statement of the objective(s) or question(s) the review addresses.	The 3 paragraph in introduction
**METHODS**			
Eligibility criteria	5	Specify the inclusion and exclusion criteria for the review and how studies were grouped for the syntheses.	2.2 Eligibility Criteria and Study Selection
Information sources	6	Specify all databases, registers, websites, organisations, reference lists and other sources searched or consulted to identify studies. Specify the date when each source was last searched or consulted.	2.1 Search Strategy and Study Sources
Search strategy	7	Present the full search strategies for all databases, registers and websites, including any filters and limits used.	2.1 and eTable S1
Selection process	8	Specify the methods used to decide whether a study met the inclusion criteria of the review, including how many reviewers screened each record and each report retrieved, whether they worked independently, and if applicable, details of automation tools used in the process.	2.2 Eligibility Criteria and Study Selection
Data collection process	9	Specify the methods used to collect data from reports, including how many reviewers collected data from each report, whether they worked independently, any processes for obtaining or confirming data from study investigators, and if applicable, details of automation tools used in the process.	2.3 Data extraction
Data items	10a	List and define all outcomes for which data were sought. Specify whether all results that were compatible with each outcome domain in each study were sought (e.g. for all measures, time points, analyses), and if not, the methods used to decide which results to collect.	2.3 Data extraction
	10b	List and define all other variables for which data were sought (e.g. participant and intervention characteristics, funding sources). Describe any assumptions made about any missing or unclear information.	2.3 Data extraction
Study risk of bias assessment	11	Specify the methods used to assess risk of bias in the included studies, including details of the tool(s) used, how many reviewers assessed each study and whether they worked independently, and if applicable, details of automation tools used in the process.	2.4 Risk of Bias
Effect measures	12	Specify for each outcome the effect measure(s) (e.g. risk ratio, mean difference) used in the synthesis or presentation of results.	2.3 Data extraction
Synthesis methods	13a	Describe the processes used to decide which studies were eligible for each synthesis (e.g. tabulating the study intervention characteristics and comparing against the planned groups for each synthesis (item #5)).	Table 1

Section and Topic	Item #	Checklist item	Location where item is reported
	13b	Describe any methods required to prepare the data for presentation or synthesis, such as handling of missing summary statistics, or data conversions.	2.5 Statistical analysis
	13c	Describe any methods used to tabulate or visually display results of individual studies and syntheses.	Figure 1, 2
	13d	Describe any methods used to synthesize results and provide a rationale for the choice(s). If meta-analysis was performed, describe the model(s), method(s) to identify the presence and extent of statistical heterogeneity, and software package(s) used.	2.5 Statistical analysis
	13e	Describe any methods used to explore possible causes of heterogeneity among study results (e.g. subgroup analysis, meta-regression).	2.5 Statistical analysis
	13f	Describe any sensitivity analyses conducted to assess robustness of the synthesized results.	2.5 Statistical analysis
Reporting bias assessment	14	Describe any methods used to assess risk of bias due to missing results in a synthesis (arising from reporting biases).	2.4 Risk of Bias
Certainty assessment	15	Describe any methods used to assess certainty (or confidence) in the body of evidence for an outcome.	2.4 Risk of Bias
**RESULTS**			
Study selection	16a	Describe the results of the search and selection process, from the number of records identified in the search to the number of studies included in the review, ideally using a flow diagram.	3.1 Search Results and eFigure S1
	16b	Cite studies that might appear to meet the inclusion criteria, but which were excluded, and explain why they were excluded.	eTable S2
Study characteristics	17	Cite each included study and present its characteristics.	3.1 Search Results
Risk of bias in studies	18	Present assessments of risk of bias for each included study.	eTable S5
Results of individual studies	19	For all outcomes, present, for each study: (a) summary statistics for each group (where appropriate) and (b) an effect estimate and its precision (e.g. confidence/credible interval), ideally using structured tables or plots.	Figure 1,2,3; eFigure S2, S3, S4, S5
Results of syntheses	20a	For each synthesis, briefly summarise the characteristics and risk of bias among contributing studies.	3.1 Search Results and 3.5 Risk of Bias Assessment
	20b	Present results of all statistical syntheses conducted. If meta-analysis was done, present for each the summary estimate and its precision (e.g. confidence/credible interval) and measures of statistical heterogeneity. If comparing groups, describe the direction of the effect.	3.2, 3.3, 3.4
	20c	Present results of all investigations of possible causes of heterogeneity among study results.	
	20d	Present results of all sensitivity analyses conducted to assess the robustness of the synthesized results.	3.6 Sensitivity analyses
Reporting biases	21	Present assessments of risk of bias due to missing results (arising from reporting biases) for each synthesis assessed.	3.5 Risk of Bias Assessment
Certainty of	22	Present assessments of certainty (or confidence) in the body of evidence for each outcome assessed.	3.5 Risk of

Section and Topic	Item #	Checklist item	Location where item is reported
evidence			Bias Assessment
**DISCUSSION**			
Discussion	23a	Provide a general interpretation of the results in the context of other evidence.	Discussion
	23b	Discuss any limitations of the evidence included in the review.	Discussion
	23c	Discuss any limitations of the review processes used.	Discussion
	23d	Discuss implications of the results for practice, policy, and future research.	Discussion
**OTHER INFORMATION**			
Registration and protocol	24a	Provide registration information for the review, including register name and registration number, or state that the review was not registered.	Methods
	24b	Indicate where the review protocol can be accessed, or state that a protocol was not prepared.	Methods
	24c	Describe and explain any amendments to information provided at registration or in the protocol.	
Support	25	Describe sources of financial or non-financial support for the review, and the role of the funders or sponsors in the review.	Funding
Competing interests	26	Declare any competing interests of review authors.	Competing interests
Availability of data, code and other materials	27	Report which of the following are publicly available and where they can be found: template data collection forms; data extracted from included studies; data used for all analyses; analytic code; any other materials used in the review.	Availability of data and materials

From: Page MJ, McKenzie JE, Bossuyt PM, Boutron I, Hoffmann TC, Mulrow CD, et al. The PRISMA 2020 statement: an updated guideline for reporting systematic reviews. BMJ 2021;372:n71. doi: 10.1136/bmj.n71

Table S9 Studies with data extracted from figures using web-based tools

Study ID	Relative abundance	Shannon	Simpson	Observed	Chao 1	ACE
Hasan 2024	—	Yes	Yes	Yes	Yes	Yes
Manzoor 2022	—	Yes	—	Yes	—	—
Zhao 2025	—	Yes	Yes	—	—	—
Dai 2016	—	Yes	—	—	—	—
Chen 2025_2	—	Yes	—	Yes	Yes	Yes
Fernández 2021	—	Yes	Yes	—	—	—
Gao 2025	—	Yes	—	—	Yes	—
Lledo 2022	Yes	Yes	Yes	Yes	Yes	Yes
Zhao 2020	—	—	—	—	Yes	—
Chopra 2024	—	—	—	Yes	Yes	Yes
Dong 2023	—	—	—	Yes	Yes	Yes
Graspeuntner 2018	—	—	Yes	—	—	—

Note. 'Yes' indicates that the data for the corresponding study were extracted from published figures using a web-based digitization tool when raw numerical values were not reported and could not be obtained from the original authors. '—' indicates that the data were directly available in the original publication or were obtained from the study authors. A complete description of the data extraction process is provided in the Methods section.

Table S10 Meta-regression analysis for α-diversity

Factors	Estimate (95%CI)	Z value	P value	k
Shannon index				
Age	-0.02 (-0.13 to 0.09)	-0.4	0.689	14
BMI	0.08 (-0.24 to 0.40)	0.507	0.612	9
Asian region	-0.33 (-1.29 to 0.63)	-0.675	0.5	16
Race category overall	Global test	—	0.811	16
ART use	Not estimable	—	—	9
Sample site overall	Global test	—	0.516	17
Observed index				
Age	7.79 (-42.14 to 57.72)	0.306	0.76	6
BMI	100.17 (70.51 to 129.82)	6.62	<0.001	3
Asian region	-69.56 (-392.41 to 253.28)	-0.422	0.673	7
Race category overall	Global test	0.673	7	—
ART use	Not estimable	—	3	—
Sample site overall	Global test	—	0.654	8
Simpson index				
Age	0.03 (-0.10 to 0.16)	0.458	0.647	9
BMI	-0.08 (-0.23 to 0.07)	-0.981	0.327	6
Asian region	-0.25 (-1.01 to 0.50)	-0.658	0.511	11
Race category overall	Global test	—	0.406	11
ART use	Not estimable	—	—	4
Sample site overall	Global test	—	0.234	12
Chao index				
Age	-15.72 (-84.90 to 53.46)	-0.445	0.656	14
BMI	54.60 (-55.88 to 165.08)	0.969	0.333	9
Asian region	-219.59 (-748.48 to 309.31)	-0.814	0.416	16
Race category overall	Global test	—	0.416	16
ART use	Not estimable	—	—	8
Sample site overall	Global test	—	0.821	17
ACE index				

Factors	Estimate (95%CI)	Z value	P value	k
Age	23.90 (-83.32 to 131.12)	0.437	0.662	6
BMI	143.58 (30.12 to 257.05)	2.48	0.013	4
Asian region	-91.94 (-746.17 to 562.28)	-0.275	0.783	7
Race category overall	Global test	0.783	7	—
ART use	Not estimable	—	3	—
Sample site overall	Global test	0.857	8	—

Note: Values are regression coefficients from univariable random-effects meta-regression models and are presented as estimates with 95% confidence intervals. Positive estimates indicate higher α-diversity values with increasing continuous moderators or relative to the reference category for categorical moderators, whereas negative estimates indicate lower values. For categorical moderators, the overall P value represents the global test for differences across categories. ART-related moderators were not estimable when there were insufficient variation or too few available studies in the included data. k indicates the number of effect sizes included in each meta-regression model. ART, assisted reproductive technology; BMI, body mass index; CI, confidence interval.
